# Dragon's Paradise Lost: Palaeobiogeography, Evolution and Extinction of the Largest-Ever Terrestrial Lizards (Varanidae)

**DOI:** 10.1371/journal.pone.0007241

**Published:** 2009-09-30

**Authors:** Scott A. Hocknull, Philip J. Piper, Gert D. van den Bergh, Rokus Awe Due, Michael J. Morwood, Iwan Kurniawan

**Affiliations:** 1 Geosciences, Queensland Museum, Brisbane, Queensland, Australia; 2 Archaeological Studies Program, University of the Philippines, Quezon City, Philippines; 3 School of Earth and Environmental Sciences, University of Wollongong, Wollongong, New South Wales, Australia; 4 Indonesian Centre for Archaeology, Jakarta, Indonesia; 5 Geological Survey of Indonesia, Bandung, Indonesia; Zoological Society of London, United Kingdom

## Abstract

**Background:**

The largest living lizard species, *Varanus komodoensis* Ouwens 1912, is vulnerable to extinction, being restricted to a few isolated islands in eastern Indonesia, between Java and Australia, where it is the dominant terrestrial carnivore. Understanding how large-bodied varanids responded to past environmental change underpins long-term management of *V. komodoensis* populations.

**Methodology/Principal Findings:**

We reconstruct the palaeobiogeography of Neogene giant varanids and identify a new (unnamed) species from the island of Timor. Our data reject the long-held perception that *V. komodoensis* became a giant because of insular evolution or as a specialist hunter of pygmy *Stegodon*. Phyletic giantism, coupled with a westward dispersal from mainland Australia, provides the most parsimonious explanation for the palaeodistribution of *V. komodoensis* and the newly identified species of giant varanid from Timor. Pliocene giant varanid fossils from Australia are morphologically referable to *V. komodoensis* suggesting an ultimate origin for *V. komodoensis* on mainland Australia (>3.8 million years ago). *Varanus komodoensis* body size has remained stable over the last 900,000 years (ka) on Flores, a time marked by major faunal turnovers, extinction of the island's megafauna, the arrival of early hominids by 880 ka, co-existence with *Homo floresiensis*, and the arrival of modern humans by 10 ka. Within the last 2000 years their populations have contracted severely.

**Conclusions/Significance:**

Giant varanids were once a ubiquitous part of Subcontinental Eurasian and Australasian faunas during the Neogene. Extinction played a pivotal role in the reduction of their ranges and diversity throughout the late Quaternary, leaving only *V. komodoensis* as an isolated long-term survivor. The events over the last two millennia now threaten its future survival.

## Introduction

Fossils of giant varanids (≥3 m Total Body Length) were first reported in the 1850s with the description of *Megalania prisca* from the Pleistocene of Australia [1], [2]. Since that time, and with the discovery of living Komodo Dragons (*V. komodoensis*) on the east Indonesian islands of Flores, Rinca and Komodo [3] considerable attention was paid in trying to understand the evolution of body size in monitor lizards [4]–[6]. Though several processes are proposed to explain the evolution of giantism in varanids, two competing hypotheses dominate the literature: autapomorphic giantism (i.e. Island Rule) and phyletic giantism (i.e. Cope's Rule) [7]. Both processes were previously invoked for the evolution of *V. komodoensis* [4], [6], [7].

It is commonly thought that *V. komodoensis* is a classic example of autapomorphic giantism having evolved large body size sometime in the past from a small-bodied ancestor that arrived on isolated Indonesian islands, which were devoid of predatory competition [3], [8]. Some proposals suggest that *V. komodoensis* attained large body size on Flores as a specialist hunter of pygmy *Stegodon* [3], [9], the only large-bodied prey inhabiting Flores throughout the middle and late Pleistocene to as recently as 12,000 years ago [10], [11]. The alternative, phyletic giantism, is supported by independent phylogenetic studies of morphology [12]–[14] and genetics [15], [16], which nest *V. komodoensis* within an Australopapuan clade of varanids containing the two large-sized living species, *V. salvadorii* and *V. varius*, and the largest of all known lizards *Megalania prisca ( = Varanus prisca*) [14]. Thus, large body size is a synapomorphy of the clade and is not an autapomorphic trait of *V. komodoensis*. The implications of the phyletic model are that: 1. The extant populations of *V. komodoensis* are relictual, having had a much wider geographic distribution in the past [17], [18]. 2. *Varanus komodoensis* arrived on Flores already large and did not evolve giantism there through the processes of insular evolution [7].

We aim to reconstruct the palaeobiogeography and geochronology of Neogene large-bodied varanids by using the fossil remains available from deposits in India, Java, Flores, Timor and Australia.

## Methods

### Morphometrics

**Figure 1:**
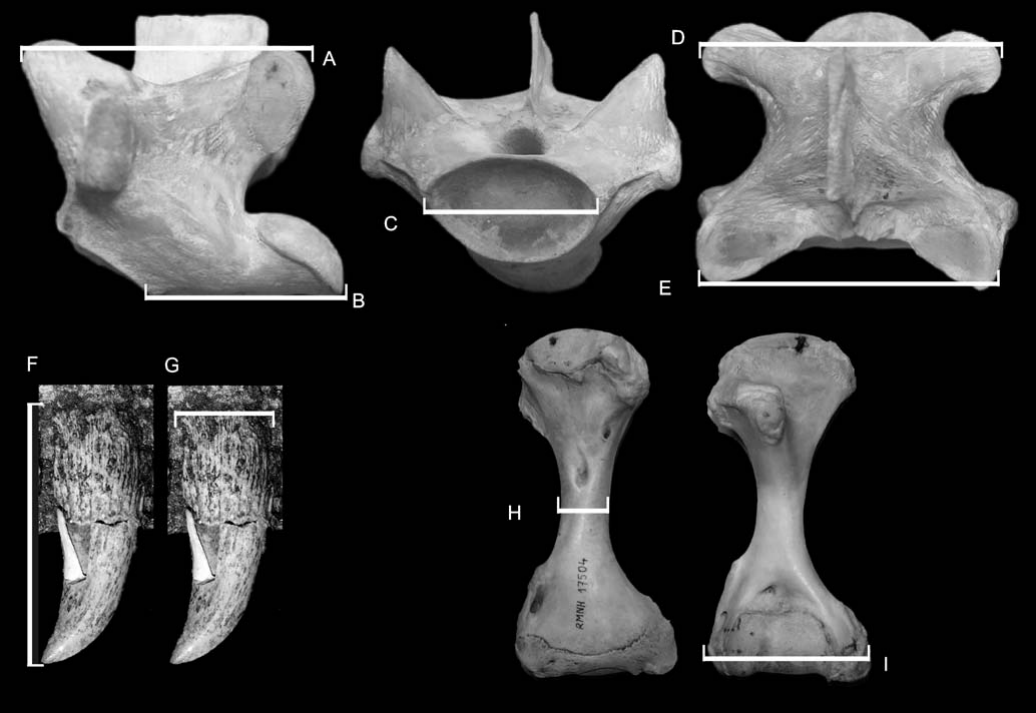
Morphometric measurements. A. Pre-postzygapophysis length. B. Centrum length. C. Cotylar width. D. Post-postzygapophysis width. E. Pre-prezygapophysis width. F. Tooth crown height. G. Tooth base length. H. Diaphysis width (humerus). I. Distal condyle width (humerus).

Five measurements were taken of fossil and modern *Varanus* cervical, dorsal, sacral and anterior caudal vertebrae (Figure 1). Measurements were undertaken using dial or digital callipers to 0.5 mm resolution. See Table S1 for specimen list and data. Measurements are in millimetres (mm) and include:

1. Prezygapophysis to postzygapophysis length (Pre-Post), measured from the anterior margin of the prezygapophyses to the posterior margin of the postzygapophyses (Figure 1, A).
2. Centrum length (CL), measured from the posterior margin of the cotyle to the posterior margin of the condyle (Figure 1, B).
3. Cotylar width (CW), measured from the left lateral margin of the cotyle to the right lateral margin of the cotyle (Figure 1, C).
4. Postzygapophysis to postzygapophysis width (Post-Post), measured from the lateral margin of the left postzygapophysis to the lateral margin of the right postzygapophysis (Figure 1, D).
5. Prezygapophysis to prezygapophysis width (Pre-Pre), measured from the lateral margin of the left prezygapophysis to the lateral margin of the right prezygapophysis (Figure 1, E).

Two measurements were taken of fossil and modern *Varanus* teeth (Figure 1).

1. Crown height, measured from the base of the tooth plicidentine to the crown tip if preserved (Figure 1, F).
2. Basal width, measured from the anterior margin to the posterior margin of the base of the tooth (Figure 1, G).

Two measurements were taken from the humerus of fossil and modern *Varanus* (Figure 1) [19].

1. Maximum diaphysis width of humeri (Figure 1, H).
2. Maximum width of the distal end (Figure 1, I).

### Capturing Maximal Size Variation

Our modern sample of *V. salvator* may not represent the maximal size limit seen in the largest *V. salvator* individuals. Our comparative sample of *V. salvator* was close to the maximal snout-vent lengths (SVL) recorded in a large sample of this taxon;, however, the largest specimen from our sample had a total length approximately 15–20% shorter than the total length of the longest recorded *V. salvator* (321 cm) [20]. Therefore, we took the measurements of our largest *V. salvator* vertebral specimens and increased them by 20%, adding these additional measurements to the main dataset. This provided a more accurate estimate of *V. salvator* maximal size limits.

### Descriptive Statistics

Bivariate plots of morphometric data were plotted to determine the position of fossil specimens in relation to the modern samples taken of *Varanus*. Convex hulls were drawn to delineate the area of maximal variation observed in the samples. Due to the different preservation states of many of the specimens, only a single measurement might be available (univariate data). For these data frequency distribution histograms or box-plots showing the median value, 25–75% quartiles and the minimal and maximal values, were produced to determine where in the range of variation the fossil specimens plotted out. Principle components analysis (PCA) was applied to analyse multivariate data; however, most multivariate analyses were uninformative due to the large amount of missing data from the fossil specimens.

Where possible statistical tests were carried out where fossil sample sizes were sufficient to return an informative result. Descriptive statistics and tests were conducted using PAST software version 1.82b [21].

## Results

### *Varanus komodoensis* Ouwens 1912

#### Australia (Early Pliocene – middle Pleistocene)

Fossil specimens from Pliocene and Pleistocene-aged sites in Australia (Table 1) were identified as belonging to *Varanus komodoensis* on the basis of the following combination of unique cranial and post-cranial characteristics. Overall similar size and proportions of all preserved skeletal elements. Maxilla contributes to the labial margin of the premaxillary-maxillary aperture (pmp). Maxillary margin of the pmp shallow. Premaxillary-maxillary suture faces antero-lingually. Angulate maxillary crest. Labio-lingually compressed, closely-set recurved and serrated dentition both on maxillae and dentaries. At least 12 tooth loci in dentary. Parietal with distinct supratemporal crests, with fronto-parietal suture interlocking. Humerus stockier than all other members of *Varanus*, except *V. prisca*.

**Table 1:** Pliocene – Pleistocene fossils from Queensland representing *Varanus komodoensis*.

Specimen	Registration	Location (Fauna)	Age	Age Reference
Cervical vertebra	QMF 23684	Bluff Downs Local Fauna, north-eastern Queensland	Early Pliocene	[32]
Dorsal vertebra	QMF 23686	Bluff Downs Local Fauna, north-eastern Queensland	Early Pliocene	[32]
Right maxilla	QMF 874	Chinchilla Sands Local Fauna, south-east Queensland	Middle Pliocene	[33]
Right maxilla (partial)	QMF 42105	Chinchilla Sands Local Fauna, south-east Queensland	Middle Pliocene	[33]
Left dentary (partial)	QMF 870+QMF 871	Chinchilla Sands Local Fauna, south-east Queensland	Middle Pliocene	[33]
Quadrate	QMF 42156	Chinchilla Sands Local Fauna, south-east Queensland	Middle Pliocene	[33]
Supraorbital	QMF 25392	Chinchilla Sands Local Fauna, south-east Queensland	Middle Pliocene	[33]
Parietal	QMF 53956	Chinchilla Sands Local Fauna, south-east Queensland	Middle Pliocene	[33]
Scapulocoracoid	QMF 866	Chinchilla Sands Local Fauna, south-east Queensland	Middle Pliocene	[33]
Left humerus (partial)	QMF 53954	Chinchilla Sands Local Fauna, south-east Queensland	Middle Pliocene	[33]
Right humerus (partial)	QMF 53955	Chinchilla Sands Local Fauna, south-east Queensland	Middle Pliocene	[33]
Vertebrae	QM Colln (numerous)	Chinchilla Sands Local Fauna, south-east Queensland	Middle Pliocene	[33]
Left maxilla (partial)	QMF 54605	Mt Etna Local Fauna, central eastern Queensland	Middle Pleistocene	[28]
Supraoccipital	QMF 54607	Mt Etna Local Fauna, central eastern Queensland	Middle Pleistocene	[28]
Quadrate (right)	QMF 54606	Mt Etna Local Fauna, central eastern Queensland	Middle Pleistocene	[28]
Tibia	QMF54608	Mt Etna Local Fauna, central eastern Queensland	Middle Pleistocene	[28]
Ulna diaphysis	QMF 54604	Mt Etna Local Fauna, central eastern Queensland	Middle Pleistocene	[28]
Dorsal vertebra	QMF 54120	Mt Etna Local Fauna, central eastern Queensland	Middle Pleistocene	[28]
Caudal vertebra	QMF 1418	Marmor Quarry, eastern Queensland	Middle Pleistocene	[34]

#### Maxillae (Figure 2, B–D and Figure 3, H–I)

Three maxillae; a near complete right maxilla (QMF 874), the anterior section of a right maxilla (QMF 42105) and the posterior portion of a left maxilla (QMF 54605) share closest morphology and size with *Varanus komodoensis*. QMF42105 represents a marginally larger individual than specimens QMF874 and QMF 54605. All three share with *V. komodoensis* closely-spaced, labio-lingually compressed, recurved dentition with finely grooved plicidentine, and serrated mesial and distal margins. The teeth are morphometrically similar to the modern *V. komodoensis* sample (Figure S1).

**Figure 2:**
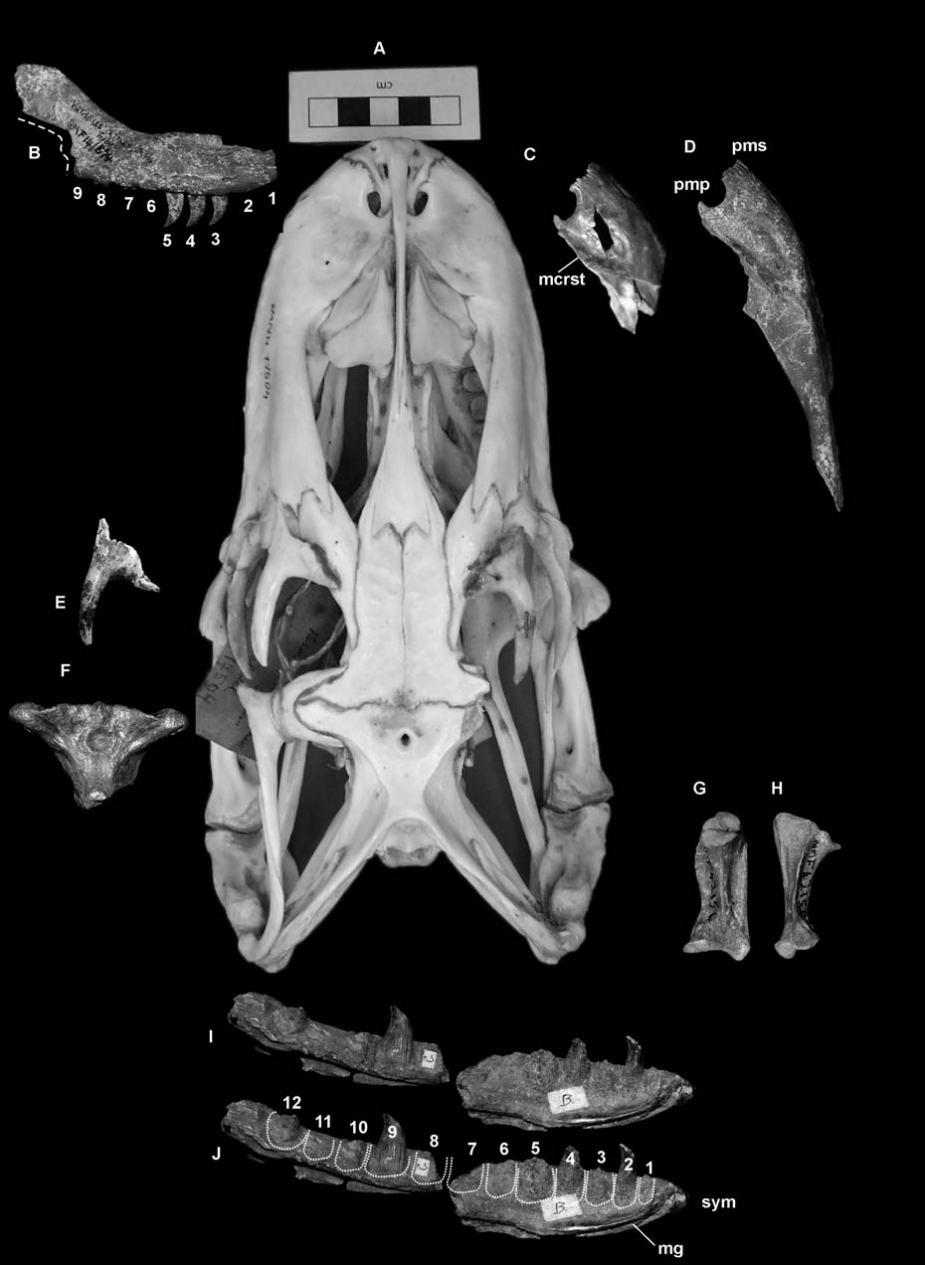
*Varanus komodoensis* (Pliocene, Australia). A. Modern *V. komodoensis* skull in dorsal view (NNM 17504). B. QMF 874, right maxilla in lateral view. C. QMF 42105, partial right maxilla in dorsal view. D. QMF 42105, right maxilla in dorsal view. E. QMF 25392, complete left supraorbital in dorsal view. F. QMF 53956, partial parietal in dorsal view. G–H. QMF 42156, right quadrate in anterior and lateral views. I–J. QMF 870+871, partial left dentary in lingual view, J illustrating the tooth loci. Abbreviations: mcrst, dorsal maxillary crest; pmp, premaxillary-maxillary aperture; pms, premaxilla-maxilla suture; sym, dentary symphysis; mg, Meckalian groove. Dashed line represents broken edge of maxilla. Scale bar = 5 cm.

**Figure 3:**
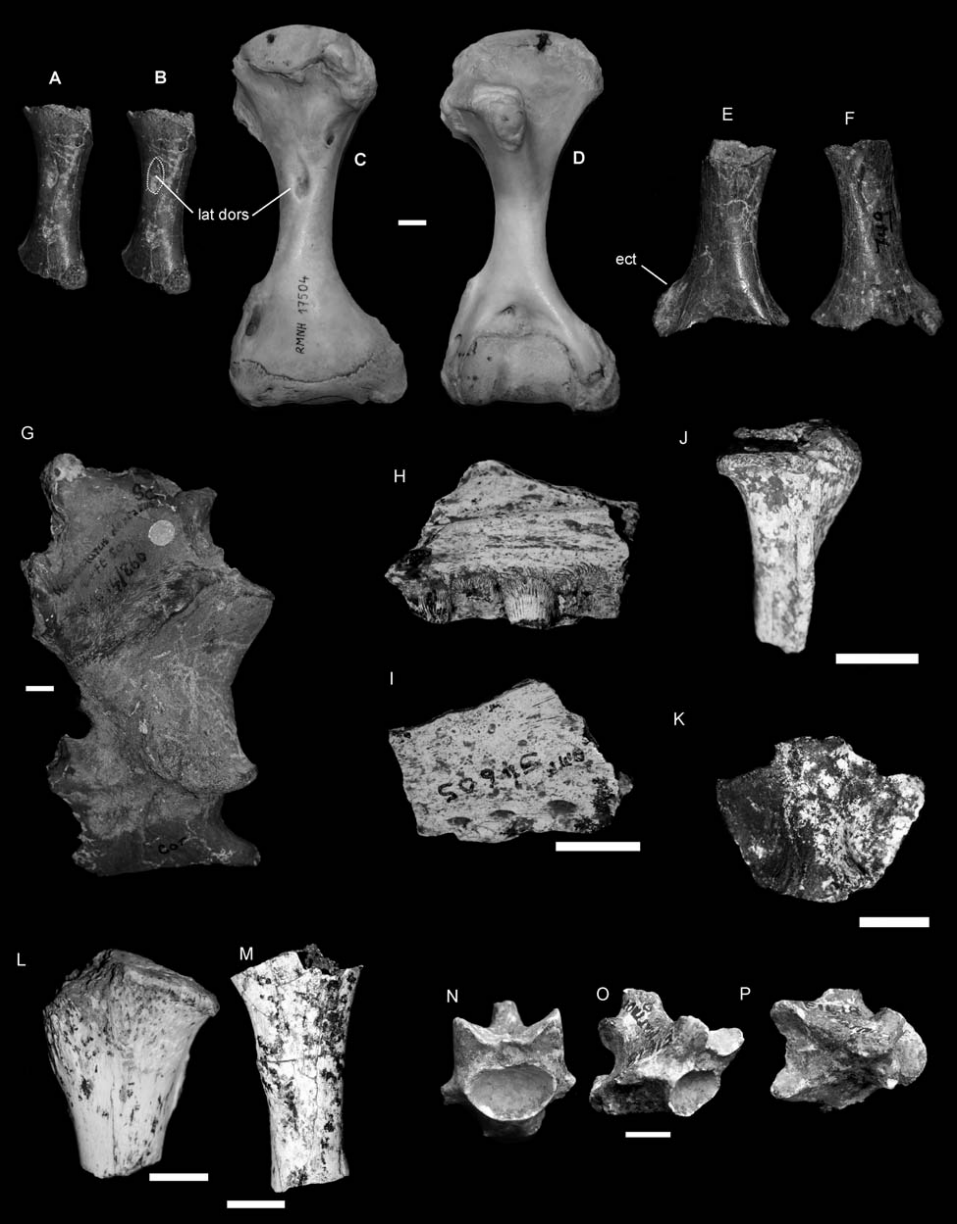
*Varanus komodoensis* (Neogene, Australia). A–B, E–G. Pliocene *V. komodoensis* (Australia)A–B. QMF 53955, partial left humerus in dorsal view showing position of insertion for the *latissimus dorsi* (lat dors). C–D. Left and right humerus of a modern *V. komodoensis* (NNM 17504). E–F. QMF 53954, partial right humerus in ventral and dorsal views, showing the position of the ectepicondyle (ect). G. QMF 866, partial scapulocoracoid. H–P. Pleistocene *V. komodoensis* (Australia). H–I. QMF 54605, partial left maxilla in lingual and labial views. J. QMF 54606, partial right quadrate in anterior view. K. QMF 54607, supraoccipital bone in posterior view. L. QMF 54608, proximal left tibia. M. QMF 54604, ulna diaphysis. N–P. QMF 1418, proximal mid-caudal in cranial, oblique lateral and dorsal views. Scale bar = 1 cm.

QMF 874 and QMF 42105 also share with *V. komodoensis* a distinct interlocking premaxillary-maxillary suture with an open premaxillary-maxillary aperture (pmp) and an angulate narial crest. The circumference of the premaxillary-maxillary aperture is made up by the premaxilla and maxilla to varying degrees in different *Varanus* groups (Figure S2). In the fossil maxillae only the posterior and labial margins of the pmp are enclosed by the maxilla, the remainder is enclosed by the premaxilla. This key feature allies the fossils to taxa where the premaxilla contributes to the anterior and lingual margin of the pmp (*V. indicus*, *V. varius* group and *V. gouldii* group).

The fossils differ morphologically from *V. indicus* by possessing an interlocking and anterolingually oriented premaxillary-maxillary suture articulation, more labially angulate maxillary crest and more recurved dentition. Morphometrically, the fossils are also much larger.

Morphologically the fossil specimens are most similar to members of the *V. varius* group and to some members of the *V. gouldii* group, in particular *V. varius*, *V. komodoensis*, *V. panoptes* and *V. mertensi* (Figure S2). The maxillary margin of the pmp in the *V. varius* group is shallower than those from the *V. gouldii* group. The pmp is similarly shallow in both fossil maxillae, allying them closer to the *V. varius* group. The only taxon present in either the *gouldii* or *varius* groups that reaches the large size of the fossils and possesses the closely-spaced, recurved dentition, is *V. komodoensis*.

#### Supraorbital (Figure 2, E)

QMF 25392 is a complete left supraorbital bone, which matches closely *V. komodoensis*, including the possession of a thick postorbital bar which projects postero-laterally, is shallowly curved and is suboval in cross-section. Proximally, the supraorbital flares in an antero-posterior direction, producing a ‘Y’ shaped bone. The dorsal surface is smooth, whilst the ventral surface preserves a rugose margin.

#### Parietal (Figure 2, F)

QMF 53956 possesses distinct dorsally expressed supratemporal crests which ally this specimen to large-sized members of the *V. varius* group (*V. salvadorii*, *V. komodoensis* and *V. prisca*). QMF 53956 is smaller than *V. prisca* with less defined crests and a broader central roof. Based on overall size, QMF 53956 is most similar to *V. komodoensis* and larger than *V. salvadorii*. It also possesses an interlocking frontal-parietal suture articulation, which is only seen in *V. komodoensis* and *V. prisca*.

#### Quadrates (Figure 2, G–H. Figure 3, J)

Two right quadrates are known, including a complete specimen (QMF 42156) and the proximal half of another (QMF 54606). Both are similar in overall morphology and size to one another and to *V. komodoensis*. In both specimens and in *V. komodoensis*, the proximal condyle is antero-posteriorly expanded into two articular facets, both rounded basins that are relatively smooth. One or two thin laminae run ventrally to the distal condyle which is medio-laterally expanded into two similar-sized condyles. A distinct medial crest originates from the midline of the proximal articular end and runs medio-distally along the medial side of the quadrate, terminating at the antero-medial corner of the disto-medial condyle. A broad, rounded and straight ridge originates at the antero-lateral corner of the proximal articular face and runs distally to the antero-lateral corner of the disto-lateral condyle.

#### Supraoccipital (Figure 3, K)

QMF 54607 is an isolated but complete supraoccipital bone from the skull of a large species of *Varanus*. In dorsal view, it is trapezoidal in shape with a ventral width wider than the dorsal width. A ridge occurs in the midline of the bone oriented dorso-ventrally and constricted toward the middle. A cup-like recess is present on the dorsal face of the bone, which would have received the *processus ascendens*, which seems to have been unossified or at least not attached to the supraoccipital (as it is in *V. priscus*). The angle of the supraoccipital in relation to the parietal, and to the paraoccipitals would be more acute than seen in *V. priscus*, similar to that of *V. komodoensis* and less so than most other species of *Varanus*.

#### Dentary (Figure 2, I–J)

QMF 870 and QMF 871 represent either a single left dentary, which is badly broken at its midline, or two separate fragments of two left dentaries. Although not noted as the same specimen, preservation and size indicates that these two specimens come from a very similar, if not the same, individual. QMF 871 is an anterior-most portion of a left dentary preserving the dentary symphysis and the first six tooth loci. The first tooth occurs just postero-dorsal of the dentary symphysis, which is rounded and bisected by the proximal origin of the Meckelian groove. The second tooth is complete and the best preserved of both specimens. The tooth is compressed labio-lingually, has a rounded distal margin and a constricted and serrate mesial cutting edge. QMF 870 is a portion of the posterior section of a left dentary, preserving five tooth loci. The dentary is missing below the dorsal margin of the Meckelian groove. The dorsal half of the posterior mental foramen can be seen in labial profile. Considered together, these two specimens indicate that the dentary preserved 12–13 tooth loci, where the teeth are closely-spaced, labio-lingually compressed, distinctly recurved and serrated. When compared to a range of *Varanus* species, it is clear that adult *V. komodoensis* possesses 12 or 13 tooth loci for each dentary; whereas other species of *Varanus* possess 11 or fewer tooth loci. *V. salvator* (10–11); *V. albigularis* (9–10); *V. indicus* (9–10); *V. varius* (9–10); *V. salvadorii* (10–11); *V. panoptes* (10–11); *V. tristis* (10–11). The only complete dentary of *V. priscus* possesses 11 tooth loci.

#### Humeri (Figure 3, A–B, E–F)

A right (QMF 53955) and a left (QMF 53954) humerus, both missing the proximal and distal-most epiphyses are of similar size and morphology to *V. komodoensis*. The humeri of *V. priscus* and *V. komodoensis* are stocky and robust when compared to humeri found in all other members of *Varanus*. Both fossil humeri indicate a stout humerus with broad proximal and distal epiphyses. When comparing the maximum diaphyseal width of the two specimens with species of extant and fossil *Varanus*, both specimens fall within the size range of *V. komodoensis* and outside that of small and large *Varanus prisca* (Figure S3).

#### Vertebrae (Figure 3, M–P. Figure 4.)

Thirty eight dorsal vertebrae were measured from two Pliocene localities, Chinchilla (n = 37) and Bluff Downs (n = 1). All of these vertebrae fell within the size range of modern *V. komodoensis* (p>0.8) (Figure S4). In all features, the fossil sample reflects directly similar features seen in *V. komodoensis*.

**Figure 4:**
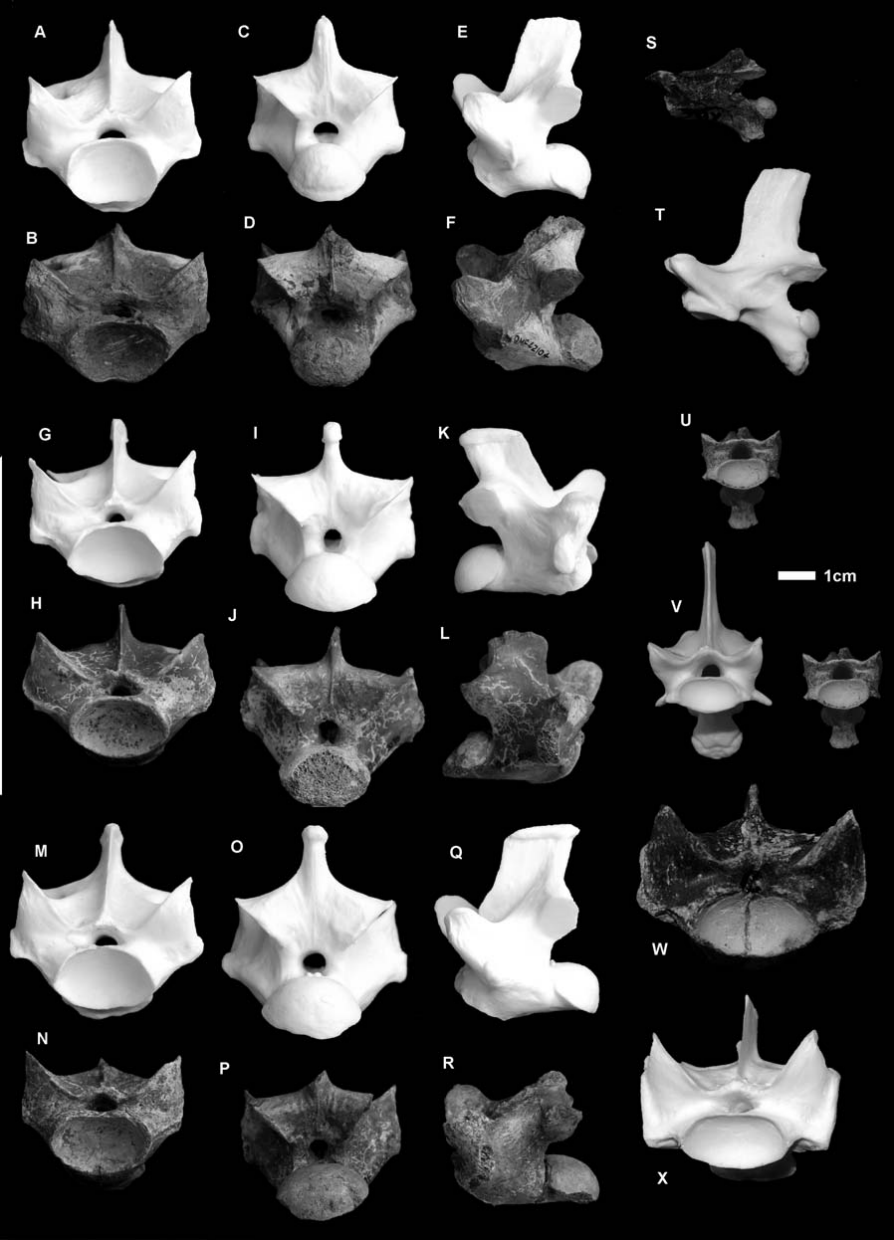
*Varanus komodoensis* (Pliocene, Australia). A–F. QMF 42104, posterior dorsal vertebra compared with modern *V. komodoensis* (white), in anterior (A–B), posterior (C–D) and left lateral (E–F) views. G–L. QMF 42096, mid-dorsal vertebra compared with modern *V. komodoensis* (white), in anterior (G–H), posterior (I–J) and right lateral (K–L) views. M–R. QMF 42102, mid-dorsal vertebra compared with modern *V. komodoensis* (white), in anterior (M–N), posterior (O–P) and left lateral (Q–R) views. S–V. QMF 23684, cervical vertebra compared with modern *V. komodoensis* (white), in left lateral (S–T) and anterior (U–V) views. W–X. QMF 23686, anterior dorsal vertebra compared with modern *V. komodoensis* (white) in anterior view. Scale bar = 1 cm.

A partial dorsal vertebra (QMF 54120) and a caudal vertebra collected from middle Pleistocene-aged sites at Mt. Etna and Marmor Quarry respectively represent a large-bodied varanid which is much smaller than *V. prisca*, but larger than any living varanid on mainland Australia and New Guinea (e.g. *Varanus giganteus*, *V. varius*, *V. salvadorii*) (Figure 4). QMF 54120 is a fragmentary dorsal vertebra, preserving the left lateral half of the cotyle and the left lateral portion of the postzygapophysis. On direct comparison with *V. komodoensis* it shares similar size and morphology. QMF1418 is a near complete proximal mid-caudal vertebra and falls within the size range of *V. komodoensis* (Figure S9).

#### Other postcranial elements (Figure 3, L–M)

In addition to the above diagnostic skeletal elements, several other postcranial remains recovered from these Pliocene and Pleistocene sites match *V. komodoensis* in over size and general morphology. These specimens include the proximal end of a left tibia (QMF 54608), the proximal end of a dorsal rib (QMF 54603), the diaphysis of an ulna (QMF 54604) and a partial scapulocoracoid (QMF866).

### Flores (early Pleistocene - Holocene)

Fossil specimens of *V. komodoensis* were recovered from the early-middle Pleistocene Ola Bula Formation in central Flores (Tangi Talo, Dhozo Dhalu) and from a late Pleistocene-Holocene cave deposit in central-western Flores (Liang Bua) [10], [22]. Fossil specimens of *V. komodoensis* include many cranial and postcranial elements (Table S1).

#### Teeth (Figure 5, E)

Twelve teeth were studied from the Pleistocene of Flores, including six isolated teeth from early Pleistocene Tangi Talo and six teeth from late Pleistocene-Holocene Liang Bua. Morphometrically these teeth fall within the size range of *V. komodoensis* (Figure S1). Morphologically, the teeth bear the unique features of being greatly recurved and compressed labio-lingually. This feature is only present in modern *V. komodoensis* and fossil specimens referable to *V. komodoensis*.

**Figure 5:**
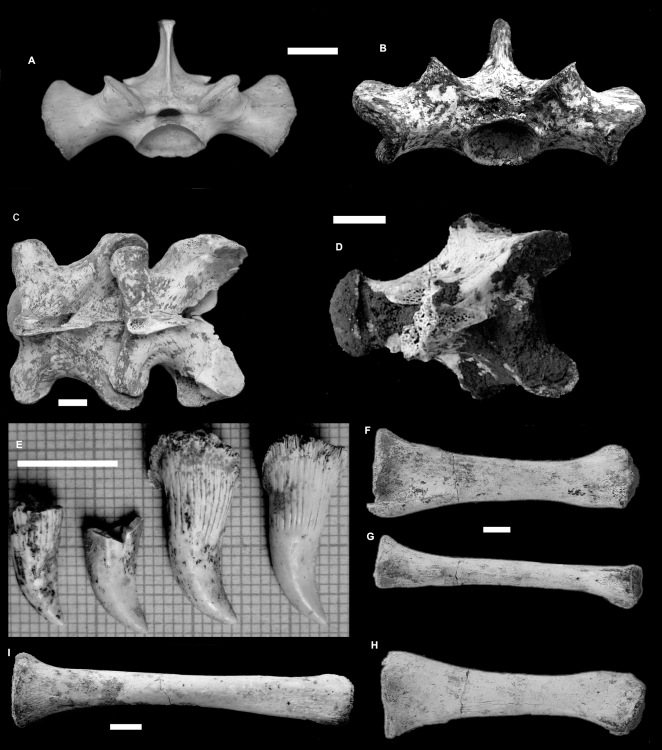
*Varanus komodoensis* (Pleistocene, Flores). A–B. Sacral vertebrae from modern (A) and fossil (LB558a) *V. komodoensis* in anterior view. C. Articulated dorsal vertebrae (LB19/20-9-04) in dorsal view. D. Cervical vertebra (LB517b) in dorsal view. E. Four isolated teeth (LB04 unreg) in lingual view. F–H. Ulna diaphysis (LB-447a/16.8.04) in medial (F), cranial (G) and lateral (H) views. I. Radius diaphysis (LB-28.7.03) in medial view. Scale bar = 1 cm.

#### Cervical vertebrae (Figure 5, D)

Four well preserved cervical specimens were used in this study, including one from early Pleistocene Tangi Talo and three from late Pleistocene-Holocene Liang Bua. The Tangi Talo specimen is only slightly larger than the modern *V. komodoensis* sample (Figure S5, A). Two Liang Bua specimens are only slightly larger and one is within the morphometric range of our modern *V. komodoensis* sample (Figure S5, B). No statistical test was conducted due to the very small sample sizes for each site (1 and 3 respectively).

#### Dorsal vertebrae (Figure 5, C)

Fifteen dorsal vertebrae were used in this study, all coming from Liang Bua. Due to the differing preservation states of each vertebra the only available measurement for all dorsal vertebrae was the cotylar width. When the Liang Bua fossil sample is compared to our sample of modern *V. komodoensis* the mean value for cotylar width is significantly different (p<0.001). When comparing the maximal size limits of our two samples the Liang Bua fossil sample is most-likely biased toward large individuals (Figure S6). Therefore, we consider the significant difference in mean cotylar width to be related to a taphonomic bias toward large individuals being preserved at the Liang Bua site, not an overall larger size. These large individuals are still within the maximal size limits of our modern sample of *V. komodoensis*. In addition, three well-preserved specimens were used in a bivariate plot of prezygapophysis – postzygapophysis (Pre-post) length over prezygapophysis – prezygapophysis (Pre-pre) width. The fossils fall within the morphometric range of modern *V. komodoensis*, with the exception of a single specimen that possesses a slightly broader pre-pre width (Figure S7). Morphologically, the vertebrae are identical to modern *V. komodoensis*.

#### Sacral vertebrae (Figure 5, B)

A single sacral vertebra from Liang Bua is directly comparable in size to *V. komodoensis* and places morphometrically within the sample of modern *V. komodoensis* (Figure S8). No statistical tests were able to be carried out due to the small sample size (n = 1).

#### Anterior caudal vertebrae

Six anterior caudal vertebrae were studied from Liang Bua, all of which fall within the morphometric and morphological variation of modern *V. komodoensis*; their mean sizes not significantly different (p>0.67) (Figure S9).

#### Humerus

A single humeral diaphysis represents *V. komodoensis* from Tangi Talo both in size and morphology. When compared to modern *V. komodoensis* and humeri from Chinchilla, the Tangi Talo specimen has a slightly broader diaphysis (Figure S3). This may simply reflect the biased preservation of larger individuals within this deposit, as is seen in the Liang Bua collection.

#### Other postcranial elements (Figure 5, H–I)

In addition to the above diagnostic specimens several other remains recovered from Liang Bua are considered to represent *V. komodoensis*, including fragments of ilia, metapodials, a phalanx, partial right mandible, and the diaphyses of an ulna and a radius (Table S1). These remains will form part of a future review of *V. komodoensis* fossils from Liang Bua.

### *Varanus* sp. cf. *V. komodoensis*

#### Java (Middle Pleistocene)

**Figure 6:**
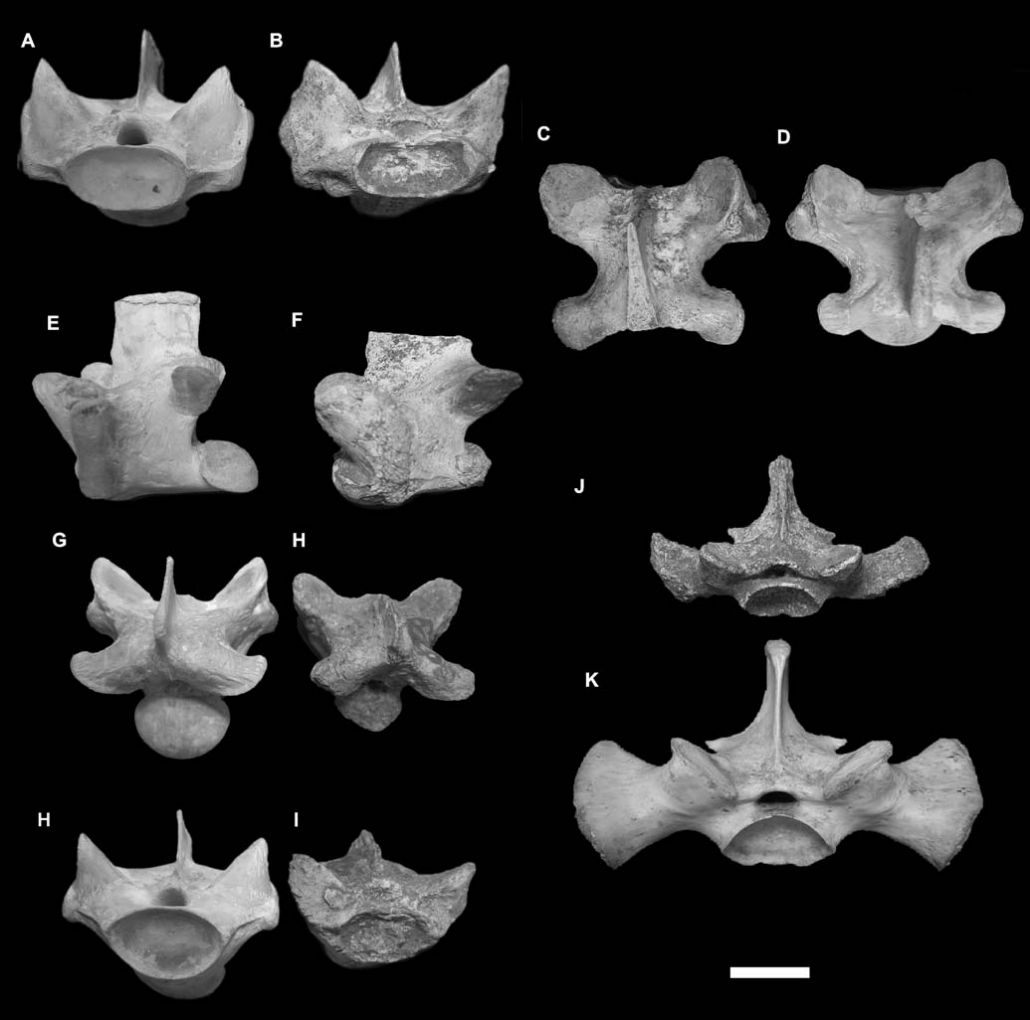
*Varanus* sp. cf. *V. komodoensis* and *V. salvator* (Pleistocene, Java). A–F. *V.* sp. cf. *V. komodoensis*. Anterior dorsal vertebra (CD 6392) compared with modern *V. komodoensis* in anterior (A–B), dorsal (C–D) and left lateral view (E–F). G–K. *V. salvator*. G–I. CD 8873, mid-dorsal vertebra, compared with modern *V. komodoensis* in dorsal (G–H) and anterior (H–I) views. J–K. CD 216, sacral vertebra, compared with modern *V. komodoensis* in anterior view. Scale bar = 1 cm.

A single anterior dorsal vertebra (CD6392) of a large-bodied varanid is recorded from the middle Pleistocene Kedung Brubus deposit (Figure 6, A–F). Morphometrically this specimen falls within the middle range of modern and fossil *V. komodoensis* and is well outside the largest *V. salvator* (+20%) sample (Figure S7). CD6392 was considered to be *V. komodoensis* [17]. It is remarkably similar to *V. komodoensis* in both size and morphology, possessing steep zygapophyses, dorsally oriented condyles, distinct precondylar constriction and an open neural canal. Although the specimen is close in morphology, assignment to *V. komodoensis* is tentative and should await more specimens for verification.

### *Varanus sivalensis* Falconer 1868

#### India, (Pliocene - early Pleistocene)

Three specimens were previously described to represent *Varanus sivalensis* [23]–[25], a distal humerus and two dorsal vertebrae (anterior and mid-dorsal vertebrae). Whether these three specimens represent a single taxon (*V. sivalensis*) will depend on the discovery of more fossil specimens referable to this taxon. The humerus is morphologically distinct from *Varanus komodoensis* to warrant its unique taxonomic status; however, the two referred dorsal vertebrae fall within the variation of modern and fossil *V. salvator*. Therefore, it is unlikely that these three specimens represent the same taxon.

#### Humerus (Figure 7, C–D)

Morphologically the humerus differs from *V. komodoensis* by features already described [12]. NHMR40816 plots in the middle range of *V. komodoensis* and outside *V. salvator*.

**Figure 7:**
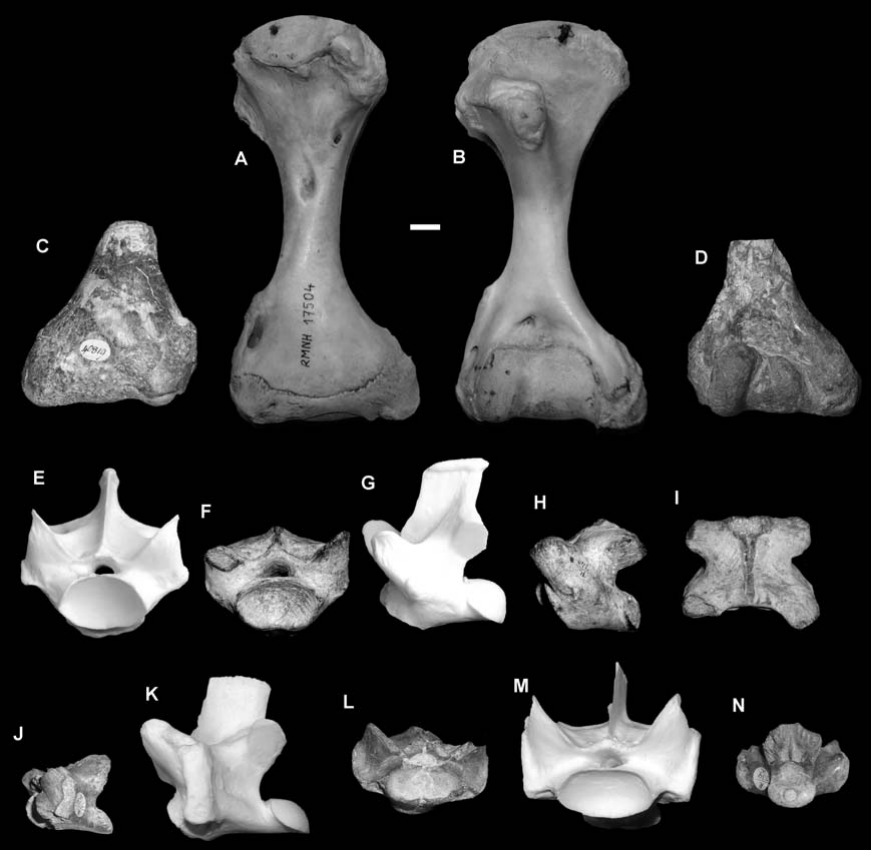
*Varanus sivalensis* (Pliocene, India). A–B. NNM 17504, modern *Varanus komodoensis* humerus. C–D. NHMR 40819, distal humerus in dorsal (C) and ventral (D) views. E–I. NHMR 740, posterior dorsal vertebra compared with modern *V. komodoensis* (white) in anterior (E–F), left lateral (G–H) and dorsal (I) views. J–N. NHMR 739, anterior dorsal compared with modern *V. komodoensis* (white) in left lateral (J–K), anterior (L–M) and posterior (N) views. Scale bar = 1 cm.

#### Dorsal vertebrae (Figure 7, F, H, I–J, L, N)

NHMR739 is an anterior dorsal vertebra and plots within the fossil sample of *V. salvator* and is only slightly larger than the extant sample of *V. salvator* (Figure S7). NHMR740 was originally designated as a cervical vertebra; however, it is clearly a mid-dorsal vertebra, lacking any features allying it to the cervical region. Morphometrically it plots within the lower range of *V. komodoensis* and outside the sample of modern *V. salvator*. The specimen does fit within the size range of fossil *V. salvator* from the early Pleistocene of Trinil, Java (Figures S6, S7). Both dorsal vertebrae are not considered to be significantly different to either the Trinil fossil sample or the modern *V. salvator* sample (Table 2). Both vertebrae are early Pleistocene in age, therefore, they match the Trinil specimens in morphology, size and age.

**Table 2:** Tukey's pairwise comparisons (ANOVA) table of fossil and modern *Varanus* dorsal vertebrae, pre-post length measurements.

	Liang Bua	Siwaliks	*V. komodoensis*	*V. salvator* (n = 29)
Trinil (n = 11)	p<0.0004*	p>0.9	p<0.002*	p>0.9
Liang Bua (n = 3)		p<0.006*	p>0.9	p<0.0002*
Siwaliks (n = 2)			p<0.03*	p>0.7
*V. komodoensis* (n = 74)				p<0.0007*

* indicates a significant difference between samples.

### *Varanus salvator* Laurenti, 1768

#### Java (Early Pleistocene)

The sample of large-bodied varanid fossils from the early Pleistocene deposits of Trinil, Java include dorsal, sacral and caudal vertebrae (Figure 6, G–K). Both the sacral and caudal vertebrae fall within the variation observed in modern *V. salvator* (Figures S8, S9). The majority of dorsal vertebrae fall within the variation of modern *V. salvator* whilst three specimens fall within the lower range of the *V. komodoensis* sample used in this study. Originally considered to be *V. komodoensis* [17], these few larger specimens are considered here to represent very large individuals of *V. salvator* even though they tend to be wider than the *V. salvator* (+20%) sample (Figure S7). This may be accounted for through allometric changes of the vertebrae in the largest individuals, where breadth of vertebra increases to a greater proportion with increased length (pers. obs.). Morphologically the vertebrae are similar to the comparative sample of *V. salvator*, being more gracile than *V. komodoensis* and *V. sivalensis*, the only two varanids closest in size to *V. salvator* and the fossil specimens. Statistically, the Trinil sample is not considered to be significantly different to the modern *V. salvator* sample, or the specimens derived from the Siwalik Hills (Table 2).

### *Varanus* sp. nov

#### Timor (Middle Pleistocene)

**Figure 8:**
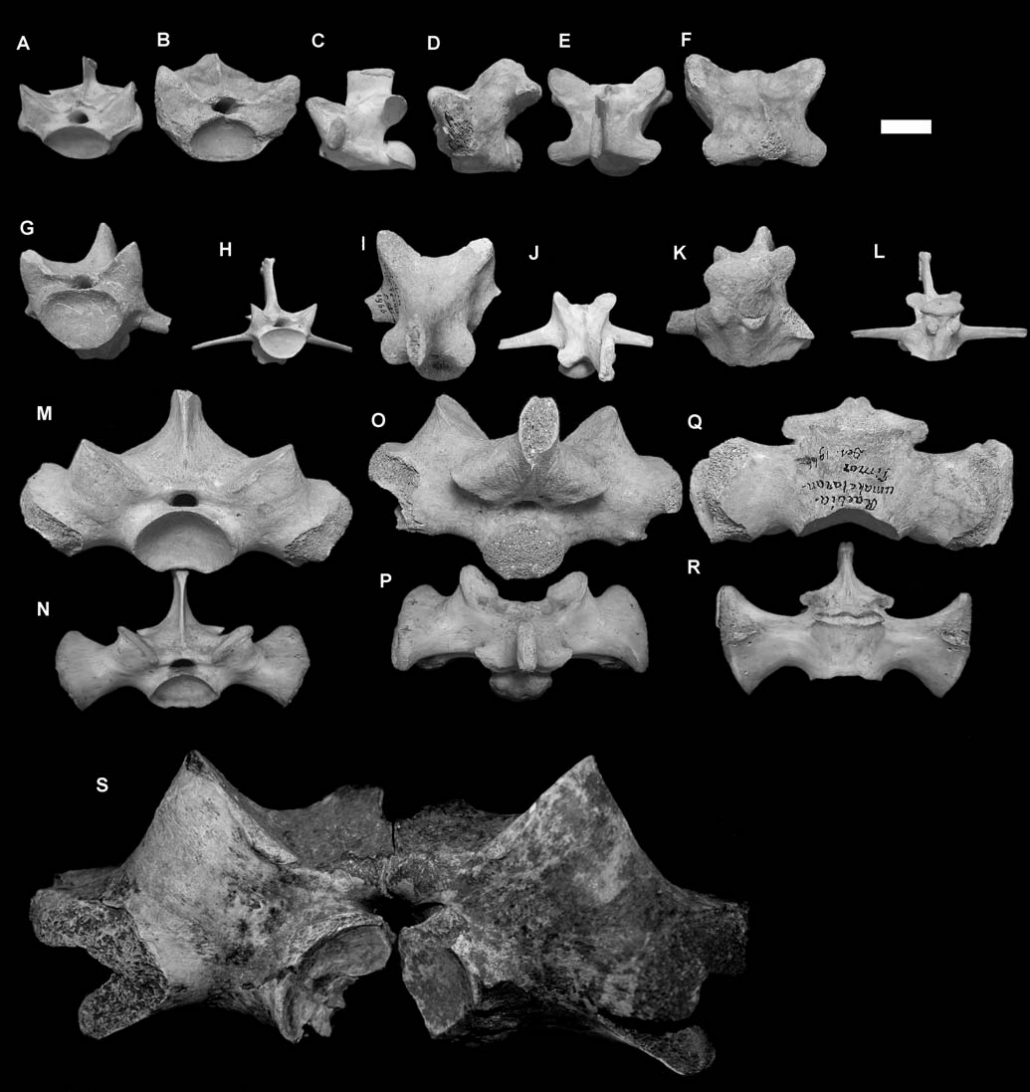
*Varanus* sp. nov. (Pleistocene, Timor). A–F. Mid-dorsal vertebra (CV Raebia 1) compared with modern *V. komodoensis* in anterior (A–B), left lateral (C–D), dorsal (E–F) views. G–L. Anterior caudal vertebra (CV Raebia 2) compared with modern *V. komodoensis* in anterior (G–H), dorsal (I–J) and oblique posterior (K–L) views. M–R. Sacral vertebra (CV Raebia 3) compared with modern *V. komodoensis* in anterior (M–N), dorsal (O–P) and ventral (Q–R) views. S. QMF 8968, sacral vertebra of *Varanus prisca* in anterior view.

Three massive varanid vertebrae are known from collections recovered from Timor, including a dorsal, sacral and anterior caudal vertebra (CV Collection, NNM). A mid-dorsal and anterior caudal were originally assigned to *V. komodoensis* [17]. The dorsal specimen falls within the upper morphometric range of *V. komodoensis*, but it possesses less vertically oriented condyles, has a reduced neural canal and is robust – features characteristic of *V. prisca* (Figures 8, S7). A sacral (not recorded previously) and an anterior caudal vertebra are morphologically similar to *V. prisca*, possessing rounded condyle-cotyles, stout transverse processes and thick cortical bone. They are both intermediate in size between *V. komodoensis* and *V. prisca* (sensu stricto) and possess zygapophyses that are at a lower angle (Figures 8, S8, S9). The combination of intermediate size and unique morphology indicate that these specimens most likely represent a new unnamed taxon of large-bodied varanid.

### Central Australia (Middle-Late Pleistocene)

#### Vertebrae

A large sample of dorsal vertebrae were measured from collections of Pleistocene varanids from central Australia. The morphometric variation encompassed by these specimens indicates the presence of a varanid intermediate in size between *V. komodoensis* and *V. prisca* (sensu stricto) (Figure S10, S11). The three samples of vertebrae were significantly different to one another with the central Australian sample showing significantly smaller size when compared to the eastern Australian *V. prisca* (sensu stricto) sample (p<0.0002) and significantly larger size when compared to modern and fossil *V. komodoensis* (p<0.004). Whether these specimens represent a diachronous sample across the middle to late Pleistocene, or a morphocline of Pleistocene giant varanids from smaller central Australian forms toward larger eastern Australian forms is yet to be determined. Regardless, these specimens indicate a giant varanid present in central Australia during the Pleistocene that resembles, at least in size, the taxon present on Timor during the middle Pleistocene.

## Discussion

Archaeological and paleontological excavations at sites in central and western Flores produced teeth and post-cranial elements of *V. komodoensis* dating from the early Pleistocene to the late Holocene (∼900–2 ka) [10], [22]. This fossil record provides an opportunity to evaluate long-term morphological and morphometric changes in *V. komodoensis* on Flores over ca. 900,000 years. Comparisons between fossil and extant *V. komodoensis* show that there are few morphometric or morphological differences between the fossil specimens and those of modern *V. komodoensis*. Therefore, maximal body size of this species remained stable for at least 900,000 years despite the fact that the biostratigraphic sequence on Flores records at least three faunal turnovers, marked by the extinction of the giant tortoise *Colossochelys* [26], two species of *Stegodon* and *Homo floresiensis*, as well as the arrival of hominins by 880 ka and modern humans by 10 ka [22]. Even in the absence of any moderately-sized prey between the extinction of *Stegodon florensis insularis* (∼12. ka) [10], [11] and the introduction of the pig from Sulawesi (∼7 ka) [10] *V. komodoensis* was able to persist on Flores. The stability of *V. komodoensis* body size over a long temporal sequence and during periods of major ecological change implies that insular evolutionary processes had limited effect, and more importantly illustrate the adaptive flexibility and resilience of a generalist carnivore, rather than a specialist predator of the island's pygmy *Stegodon*.

So, if *V. komodoensis* did not evolve on an isolated island in Wallacea, from where did it disperse? India and Australia are the only regions that preserve a giant varanid fossil record older than 900 ka, and are the only identifiable sources for large-bodied *Varanus* [2]. The oldest recorded large-bodied *Varanus* from both regions occur in the Pliocene, suggesting a relatively synchronous yet independent evolution of lizard giantism. In India large-bodied varanid fossils are rare, being represented by two vertebrae and a partial humerus, each assigned to the extinct *V. sivalensis* [24]. Both vertebrae probably represent *Varanus salvator*. The humerus is of similar size to *V. komodoensis* but differs morphologically [12]. The absence of *V. sivalensis* from younger deposits at the same locales suggests that large-bodied varanids were either very rare or more likely extinct on the Indian sub-continent by the end of the early Pleistocene. Therefore, based on both morphology and chronology, *V. sivalensis* is an unlikely source for *V. komodoensis* on Flores. *Varanus sivalensis* is associated with a Late Pliocene Siwalik fauna that includes diverse mammalian megafauna, including the placental carnivores *Crocuta*, *Hyaena* and *Panthera* [27]. This record alone demonstrates that varanids can evolve giantism on continental landmasses with competition from large placental carnivores.

Varanids appear in the Australian fossil record by the Miocene and possess a more or less continuous record of large-bodied forms from the early Pliocene (∼3.8 mya) through to the late Pleistocene [2]. Varanids most likely dispersed eastward from Asia to Australia, then radiated to produce a clade containing *V. komodoensis* [15], [16], [18]. Although the taxonomy of the Australian Miocene-Pleistocene varanids remains largely unresolved [2], it is most likely that they are contained within this monophyletic clade. There are at least three giant varanid taxa present in Australia during the Neogene, including one species from the Pliocene, one from the Pleistocene of central Australia and *Varanus prisca* (sensu stricto) from the middle-late Pleistocene. On the basis of both size and a unique combination of morphological features shared only with *V. komodoensis* the Pliocene taxon is here considered to be conspecific with *V. komodoensis*. Newly recovered large-bodied varanid fossils from middle Pleistocene [28] deposits in north-eastern Australia are also referable to *V. komodoensis*, demonstrating the longevity of *Varanus* komodoensis on mainland Australia and the coexistence of two giant varanids, *V. prisca* and *V. komodoensis*. In combination, the evidence from the fossil record as well as the morphological and molecular phylogenetic studies clearly supports Australia as the ancestral source for *V. komodoensis*.

Large-bodied varanid fossils were previously recovered from two middle Pleistocene sites along the Solo River in Java, west of the Wallace Line - the Trinil (∼900 ka) and Kedung Brubus (800–700 ka) Faunas [22]. Although large, the Trinil vertebrae fall closest to the variation of modern *V. salvator*, with a few specimens comparable in size with the smallest modern *V. komodoensis*. These few larger specimens, considered previously to be *V. komodoensis* [17], more likely represent very large individuals of *V. salvator*. A single vertebra from the younger Kedung Brubus site is much bigger, comparable closely in both size and morphology with large *V. komodoensis*. We therefore conclude that it is likely that *V. komodoensis*, having reached Flores by the early Pleistocene, dispersed westward, across Wallace's Line to arrive in Java sometime during the middle Pleistocene.

Differential timing for the initial appearance of Komodo dragon in Australia, Flores and Java, therefore indicates that *V. komodoensis* dispersed from east to west, perhaps reaching Java during a period of lowered sea-level. At the time of Kedung Brubus, Java was part of the Asian mainland, and the fauna included large placental carnivores such as *Panthera* and *Hyaena* [22], further illustrating the ability of giant varanids to exist as part of a continental, placental-dominated fauna. There is currently no evidence that giant varanids survived on Java beyond the middle Pleistocene.

Further support for the westward dispersal of giant varanids comes from Timor, an island between Flores and Australia. Three vertebral specimens from Raebia in the Atambua Basin, central Timor, represent a new unnamed species of giant varanid intermediate in size between *V. komodoensis* and *V. prisca* (*sensu stricto*). The Timor specimens were derived from the uppermost part of the folded, regressive Noele Formation, of which the marine part correlates with planktonic foraminifera zones N18-N22 [29], [30]. This suggests that the specimens are at least middle Pleistocene in age. Pleistocene varanid fossils from central Australia, usually identified as *V. prisca*, are also intermediate in size between *V. komodoensis* and *V. prisca* (*sensu stricto*), and may represent the same intermediate taxon present in Timor. Formal description of the new Timor-Australian varanid waits until more diagnostic specimens are available.

### Conclusion

The fossil record suggests that giant varanids evolved independently on mainland Asia and the island-continent of Australia during the Pliocene, alongside large-bodied mammalian carnivores. Only the Indonesian-Australian giant varanids appear to have survived beyond the early Pleistocene.

**Figure 9:**
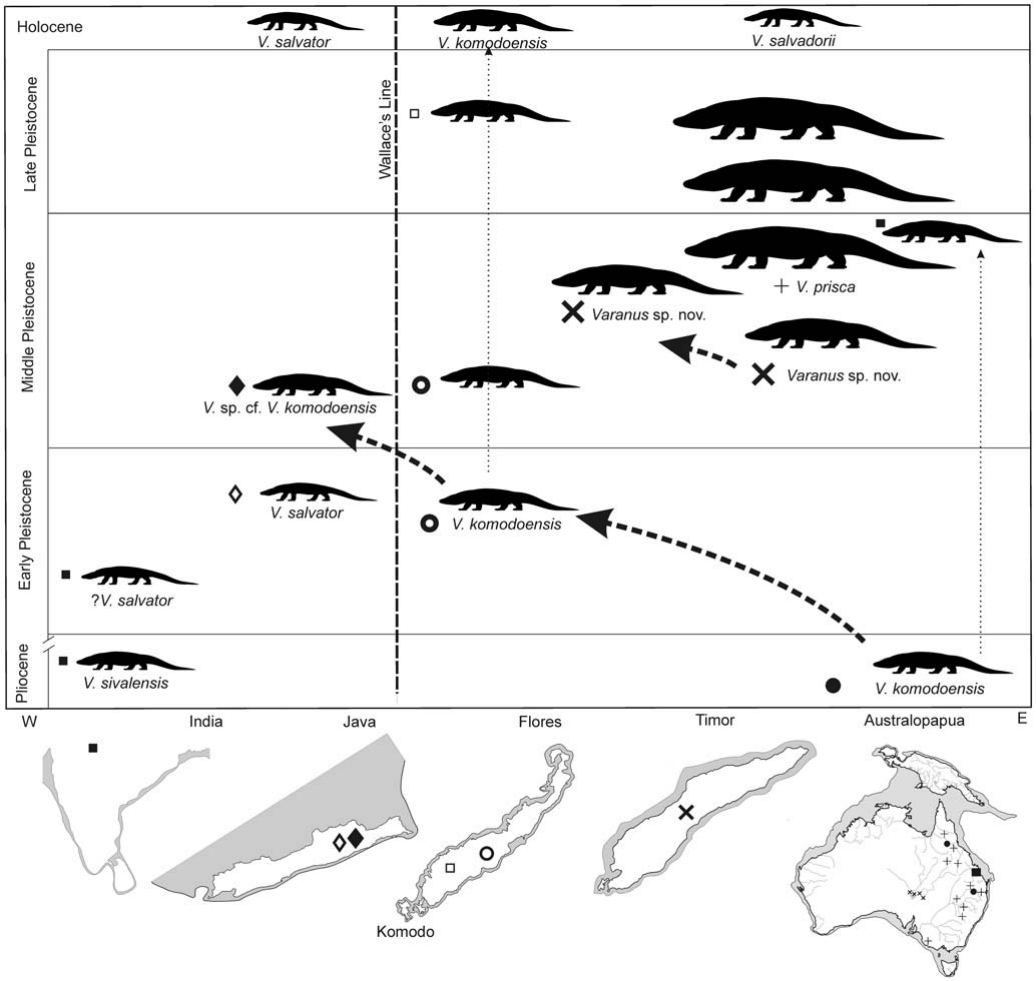
Palaeogeography and chronology of giant varanids. Schematic diagram illustrating the proposed taxonomy, chronology and dispersal sequence of giant varanids from mainland Australia to the Indonesian islands of Timor, Flores and Java during the Pliocene-Pleistocene.

We conclude that *V. komodoensis* evolved in Australia by the early Pliocene and dispersed west as far as Flores by 900 ka and Java by 800−700 ka. It is likely that the Timor varanid represents another large-bodied varanid lineage, attaining a larger body size than that of *V. komodoensis*, having evolved on mainland Australia and dispersed west to Timor. Continuing along this same evolutionary trajectory, *Varanus prisca*, reached gigantic proportions by the late-Middle Pleistocene, but was extinct in Australia by the end of the Pleistocene (Figure 9).

We conclude that *V. komodoensis* is the last of a clade of giant varanids that was once a ubiquitous part of Australasia, distributed from Australia across Wallacea, as far as continental Asia (Java). There is now only a relict population on Flores and a few small adjacent islands. Komodo dragon distribution has also retracted significantly on Flores itself; being present at Liang Bua in the uplands of West Flores until ∼2 ka, but now only occurring in isolated habitats along the northern and western coastal lowlands [3], [31]. The retraction is likely due to habitat loss and persecution by modern humans over the last few millennia and emphasizes the continuing threat of extinction to this, the last of the giant varanids.

## Supporting Information

Figure S1 Histogram of tooth base length measurements for modern (A–B), Pleistocene (A) and Pliocene (B) *V. komodoensis*. Tangi Talo (n = 4), Liang Bua (n = 5), Chinchilla (n = 5) and *V. komodoensis* (n = 68). Measurements in mm.
(0.23 MB DOC)

Figure S2 Morphological comparisons between Indo-Asian and Indo-Australian varanid maxillae based on the phylogenetic reconstruction of Ast (2001). *Varanus varius* group with fossil specimens for comparison (to scale with *V. komodoensis*).
(4.69 MB DOC)

Figure S3 Histogram of humerus maximum diaphysis width with normal curve fitted to *Varanus* sample. *Varanus* spp. (n = 71), *Varanus komodoensis* (n = 18) (see Hutchinson & Reed (2005) for taxa used). Measurements in mm.
(0.14 MB DOC)

Figure S4 Histogram of dorsal vertebrae pre-post measurements with normal curve fitted. *Varanus komodoensis* modern (n = 100), Pliocene (Chinchilla & Bluff Downs) (n = 38). Measurements in mm.
(0.19 MB DOC)

Figure S5 Measurements of varanid cervical vertebrae. A. Bivariate Plot of pre-pre length vs pre-post length. B. Bivariate Plot of cotylar width vs centrum length. Convex hulls applied to show limits of sample variation. Measurements in mm.
(0.11 MB DOC)

Figure S6 Box-plot of dorsal vertebrae cotylar width measurements. *Varanus salvator* (n = 24), Trinil (n = 15), *Varanus sivalensis* (n = 2), modern *Varanus komodoensis* (n = 112). Liang Bua (n = 16). Measurements in mm.
(0.05 MB DOC)

Figure S7 Measurements of varanid dorsal vertebrae. Bivariate Plot of pre-pre length vs pre-post length. Convex hulls applied to show limits of sample variation. Measurements in mm.
(0.09 MB DOC)

Figure S8 Measurements of varanid sacral vertebrae. A. Bivariate plot of pre-pre length vs pre-post length. Convex hulls applied to show limits of sample variation. B. Box-plot of sacral vertebrae cotylar width measurements. *Varanus salvator* (n = 10), Trinil (n = 2), *Varanus komodoensis* (n = 9), *V. prisca* (n = 4). Measurements in mm.
(0.10 MB DOC)

Figure S9 Box-plot of caudal vertebrae prezygapophysis-postzygapophysis length measurements. *Varanus salvator* (n = 9), *Varanus komodoensis* (n = 24), Liang Bua (n = 4), *V. prisca* (n = 8). Measurements in mm.
(0.06 MB DOC)

Figure S10 Box plot of dorsal vertebra pre-postzygapophysis length for *V. prisca* (n = 53), *Varanus* sp. nov. (n = 11) and modern *V. komodoensis* (n = 32). Measurements in mm.
(0.04 MB DOC)

Figure S11 Measurements of varanid dorsal vertebrae. Bivariate plot of pre-pre length vs pre-post length. Convex hulls applied to show limits of sample variation. Measurements in mm.
(0.07 MB DOC)

Table S1 Specimens used in this study.
(0.28 MB DOC)
