# Supplementary material for: Dragon's Paradise Lost: Palaeobiogeography, Evolution and Extinction of the Largest-Ever Terrestrial Lizards (Varanidae)
Source: PLoS One. 2009 Sep 30;4(9):e7241. doi: 10.1371/journal.pone.0007241 (PMC2748693; doi:10.1371/journal.pone.0007241)
Supplement: Figure S1 — Histogram of tooth base length measurements for modern (A–B), Pleistocene (A) and Pliocene (B) V. komodoensis. Tangi Talo (n = 4), Liang Bua (n = 5), Chinchilla (n = 5) and V. komodoensis (n = 68). Measurements in mm. (0.23 MB DOC) [file pone.0007241.s001.doc]

Figure S1.


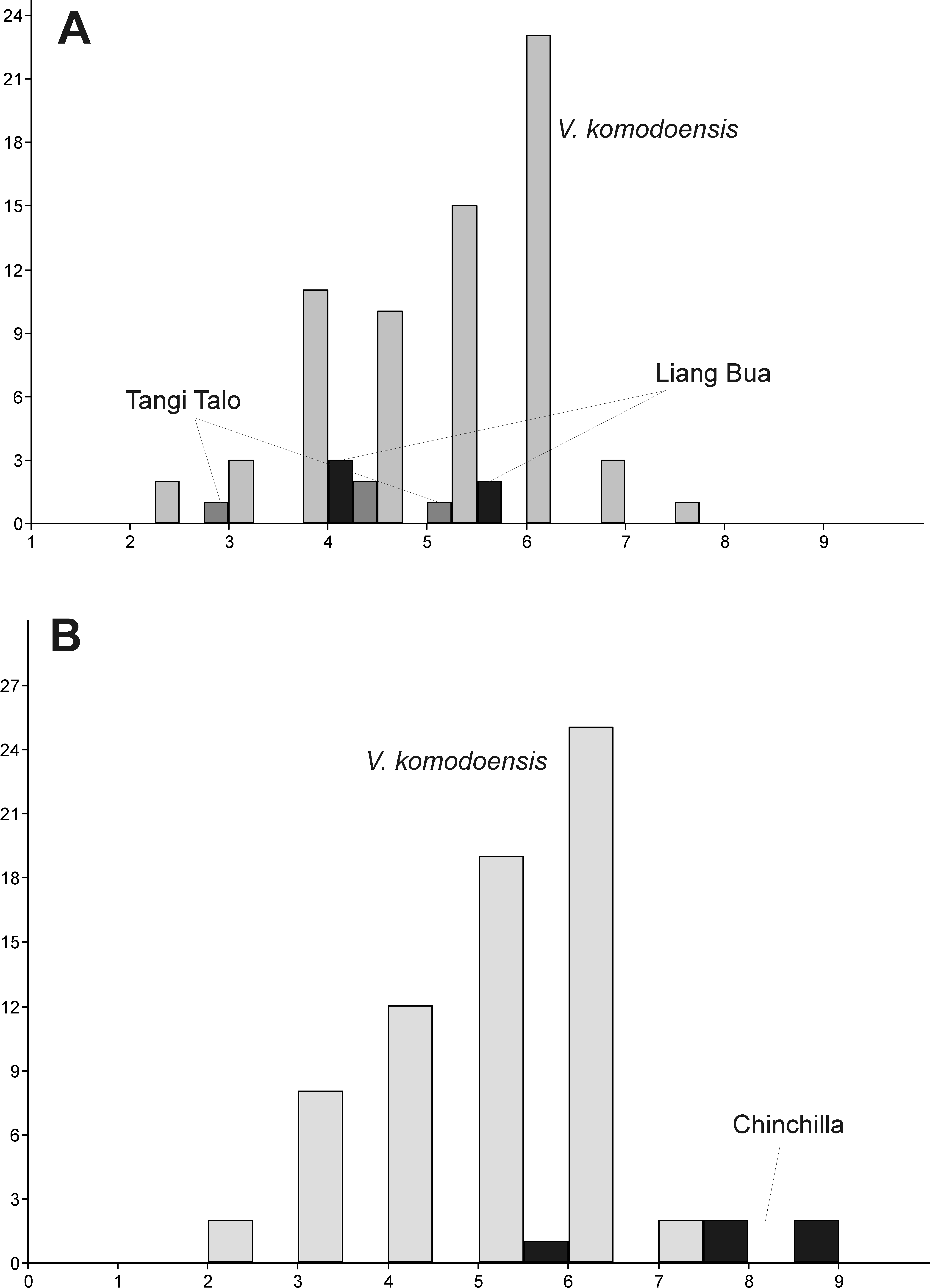


**Figure S1.** Histogram of tooth base length measurements for modern (A-B), Pleistocene (A) and Pliocene (B) *V. komodoensis.* Tangi Talo (n = 4), Liang Bua (n = 5), Chinchilla (n = 5) and *V. komodoensis* (n = 68). Measurements in mm.
