# Supplementary material for: Dragon's Paradise Lost: Palaeobiogeography, Evolution and Extinction of the Largest-Ever Terrestrial Lizards (Varanidae)
Source: PLoS One. 2009 Sep 30;4(9):e7241. doi: 10.1371/journal.pone.0007241 (PMC2748693; doi:10.1371/journal.pone.0007241)
Supplement: Figure S2 — Morphological comparisons between Indo-Asian and Indo-Australian varanid maxillae based on the phylogenetic reconstruction of Ast (2001). Varanus varius group with fossil specimens for comparison (to scale with V. komodoensis). (4.69 MB DOC) [file pone.0007241.s002.doc]

Figure S2.


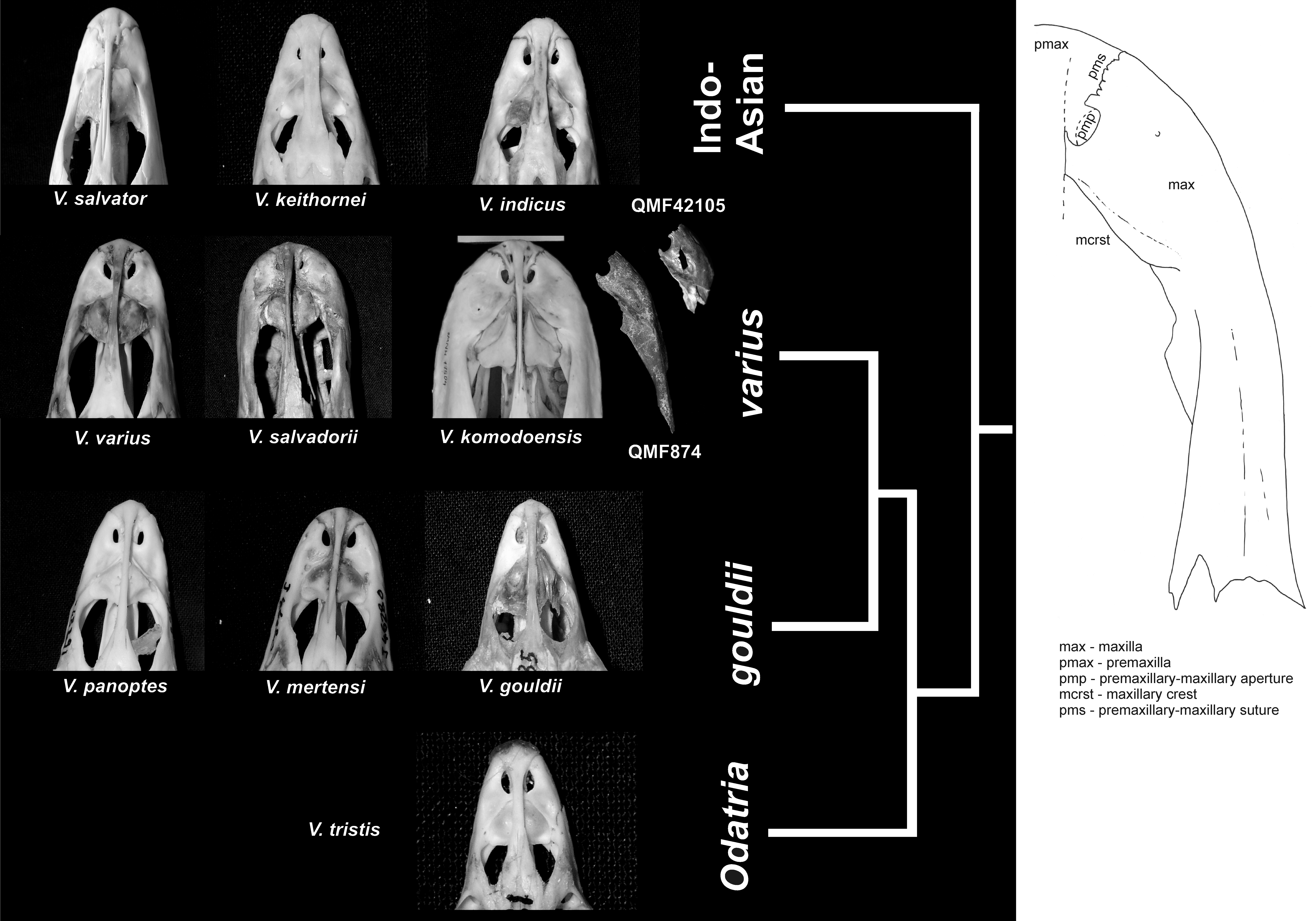


**Figure S2.** Morphological comparisons between Indo-Asian and Indo-Australian varanid maxillae based on the phylogenetic reconstruction of Ast (2001). *Varanus varius* group with fossil specimens for comparison (to scale with *V. komodoensis*).

Ast, J.C. 2001. Mitochondrial DNA Evidence and Evolution in Varanoidea (Squamata). *Cladistics*, 17, 211–226.
