# Supplementary material for: Dragon's Paradise Lost: Palaeobiogeography, Evolution and Extinction of the Largest-Ever Terrestrial Lizards (Varanidae)
Source: PLoS One. 2009 Sep 30;4(9):e7241. doi: 10.1371/journal.pone.0007241 (PMC2748693; doi:10.1371/journal.pone.0007241)
Supplement: Figure S3 — Histogram of humerus maximum diaphysis width with normal curve fitted to Varanus sample. Varanus spp. (n = 71), Varanus komodoensis (n = 18) (see Hutchinson & Reed (2005) for taxa used). Measurements in mm. (0.14 MB DOC) [file pone.0007241.s003.doc]

Figure S3.


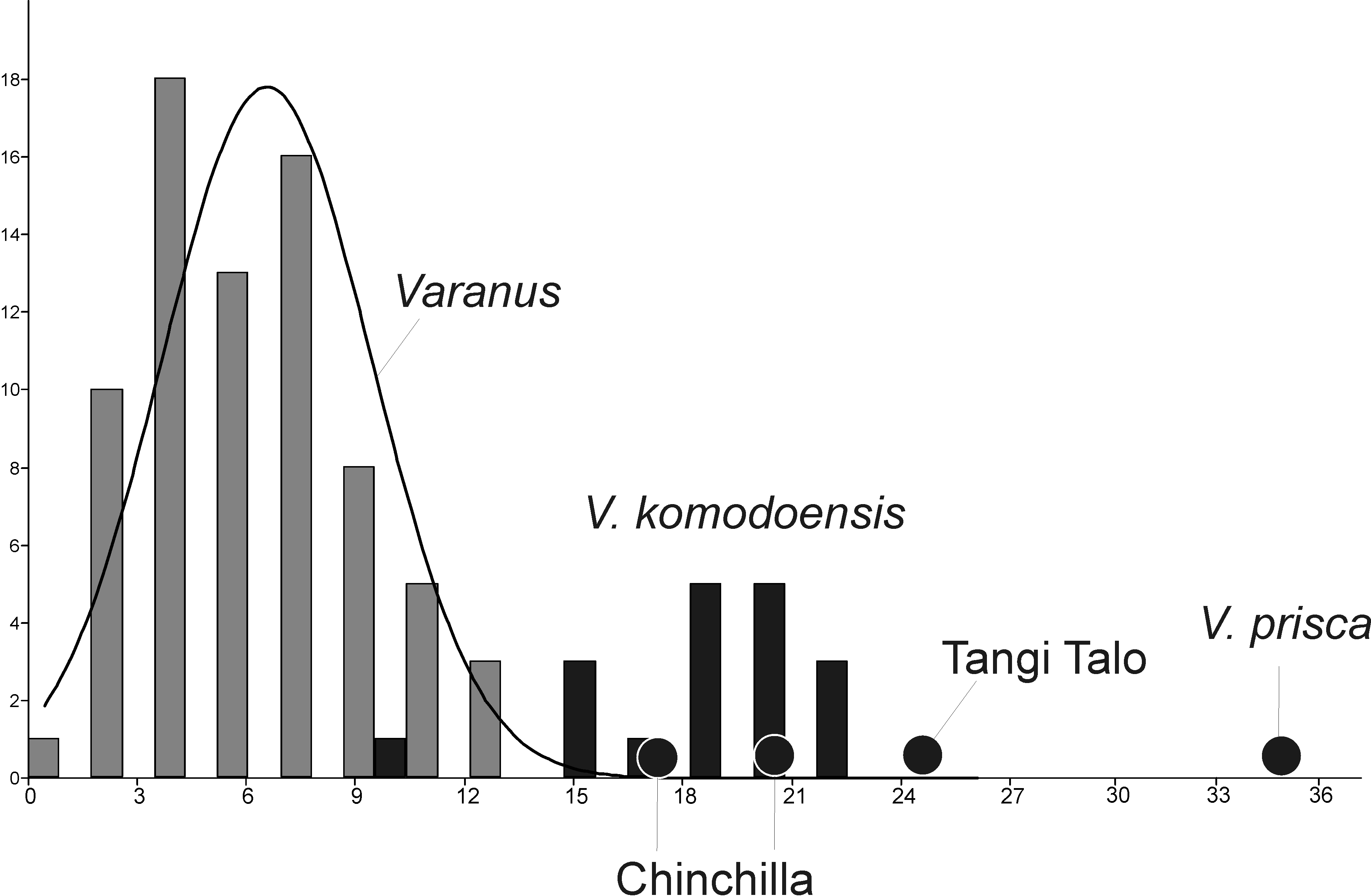


Figure S3. Histogram of humerus maximum diaphysis width with normal curve fitted to *Varanus* sample. *Varanus* spp. (n = 71), *Varanus komodoensis* (n = 18) (see Hutchinson & Reed (2005) for taxa used) . Measurements in mm.
