# Supplementary material for: Dragon's Paradise Lost: Palaeobiogeography, Evolution and Extinction of the Largest-Ever Terrestrial Lizards (Varanidae)
Source: PLoS One. 2009 Sep 30;4(9):e7241. doi: 10.1371/journal.pone.0007241 (PMC2748693; doi:10.1371/journal.pone.0007241)
Supplement: Figure S4 — Histogram of dorsal vertebrae pre-post measurements with normal curve fitted. Varanus komodoensis modern (n = 100), Pliocene (Chinchilla & Bluff Downs) (n = 38). Measurements in mm. (0.19 MB DOC) [file pone.0007241.s004.doc]

Figure S4


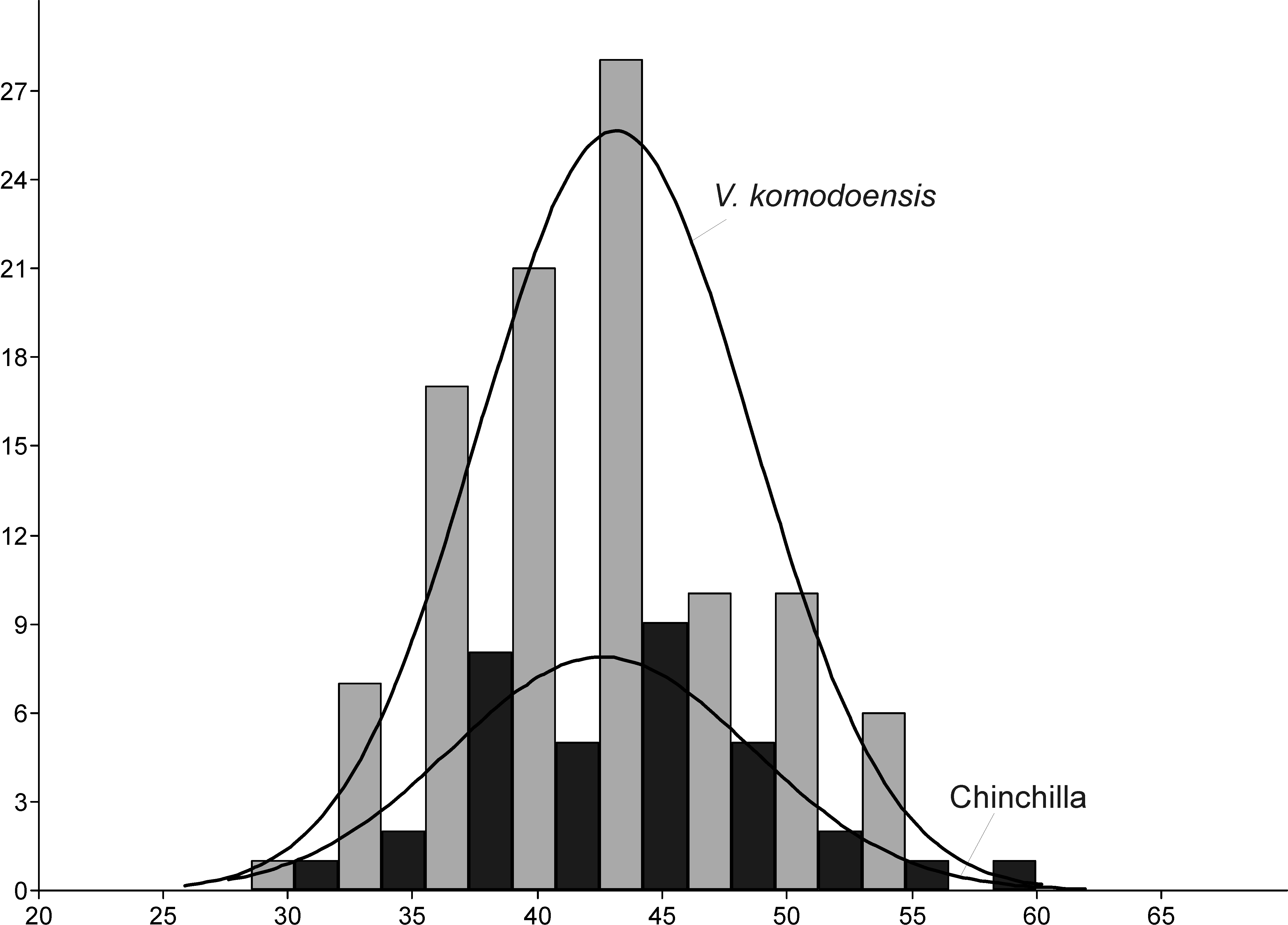


Figure S4. Histogram of dorsal vertebrae pre-post measurements with normal curve fitted*. Varanus komodoensis* modern (n = 100), Pliocene (Chinchilla & Bluff Downs) (n = 38). Measurements in mm.
