# Supplementary material for: Dragon's Paradise Lost: Palaeobiogeography, Evolution and Extinction of the Largest-Ever Terrestrial Lizards (Varanidae)
Source: PLoS One. 2009 Sep 30;4(9):e7241. doi: 10.1371/journal.pone.0007241 (PMC2748693; doi:10.1371/journal.pone.0007241)
Supplement: Figure S5 — Measurements of varanid cervical vertebrae. A. Bivariate Plot of pre-pre length vs pre-post length. B. Bivariate Plot of cotylar width vs centrum length. Convex hulls applied to show limits of sample variation. Measurements in mm. (0.11 MB DOC) [file pone.0007241.s005.doc]

Figure S5.


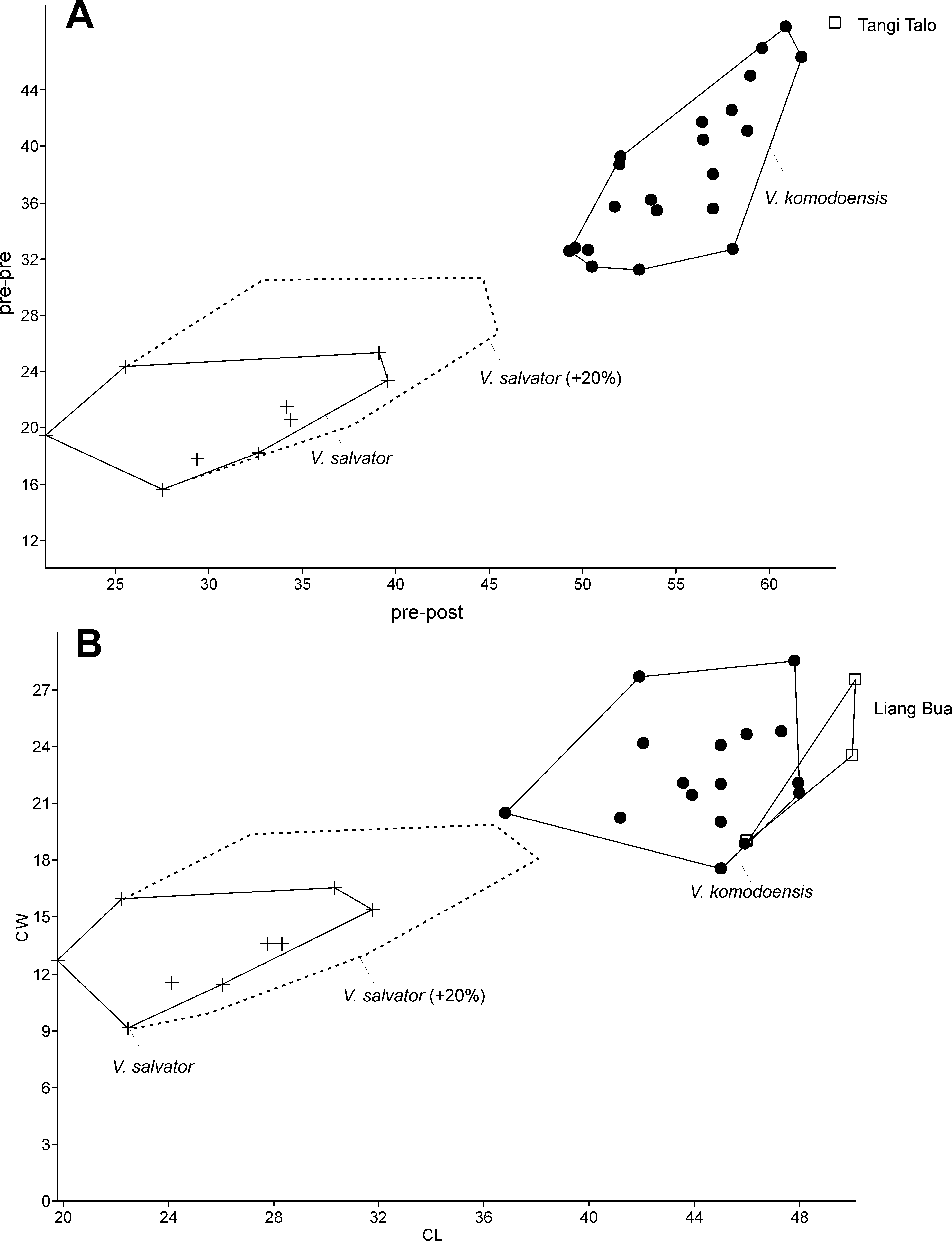


Figure S5. Measurements of varanid cervical vertebrae. A. Bivariate Plot of pre-pre length vs pre-post length. B. Bivariate Plot of cotylar width vs centrum length. Convex hulls applied to show limits of sample variation. Measurements in mm.
