# Supplementary material for: Dragon's Paradise Lost: Palaeobiogeography, Evolution and Extinction of the Largest-Ever Terrestrial Lizards (Varanidae)
Source: PLoS One. 2009 Sep 30;4(9):e7241. doi: 10.1371/journal.pone.0007241 (PMC2748693; doi:10.1371/journal.pone.0007241)
Supplement: Figure S6 — Box-plot of dorsal vertebrae cotylar width measurements. Varanus salvator (n = 24), Trinil (n = 15), Varanus sivalensis (n = 2), modern Varanus komodoensis (n = 112). Liang Bua (n = 16). Measurements in mm. (0.05 MB DOC) [file pone.0007241.s006.doc]

Figure S6.


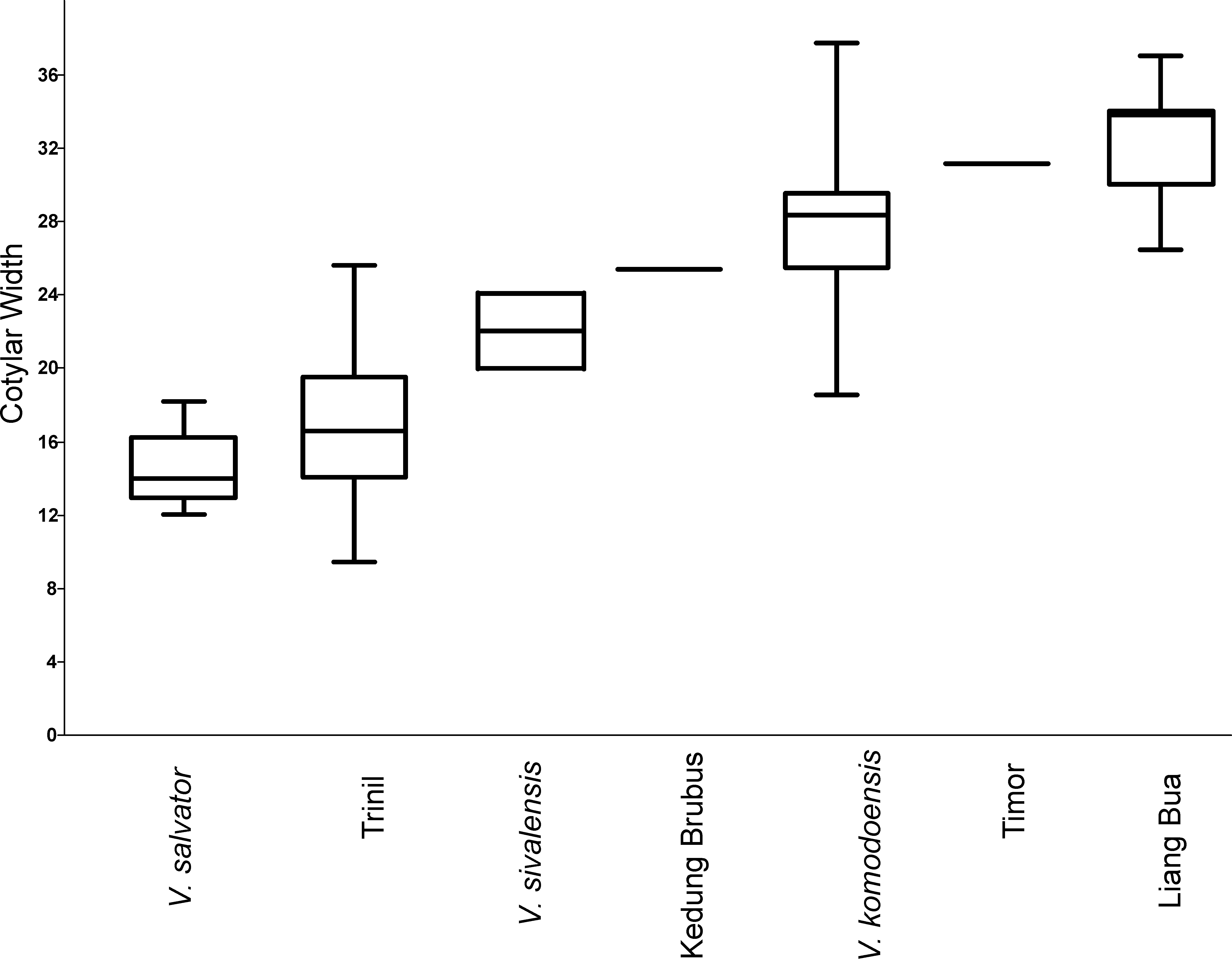


Figure S6. Box-plot of dorsal vertebrae cotylar width measurements. *Varanus salvator* (n = 24), Trinil (n = 15), *Varanus sivalensis* (n=2), modern *Varanus komodoensis* (n = 112). Liang Bua (n = 16). Measurements in mm.
