# Supplementary material for: Dragon's Paradise Lost: Palaeobiogeography, Evolution and Extinction of the Largest-Ever Terrestrial Lizards (Varanidae)
Source: PLoS One. 2009 Sep 30;4(9):e7241. doi: 10.1371/journal.pone.0007241 (PMC2748693; doi:10.1371/journal.pone.0007241)
Supplement: Figure S8 — Measurements of varanid sacral vertebrae. A. Bivariate plot of pre-pre length vs pre-post length. Convex hulls applied to show limits of sample variation. B. Box-plot of sacral vertebrae cotylar width measurements. Varanus salvator (n = 10), Trinil (n = 2), Varanus komodoensis (n = 9), V. prisca (n = 4). Measurements in mm. (0.10 MB DOC) [file pone.0007241.s008.doc]

Figure S8.

A.


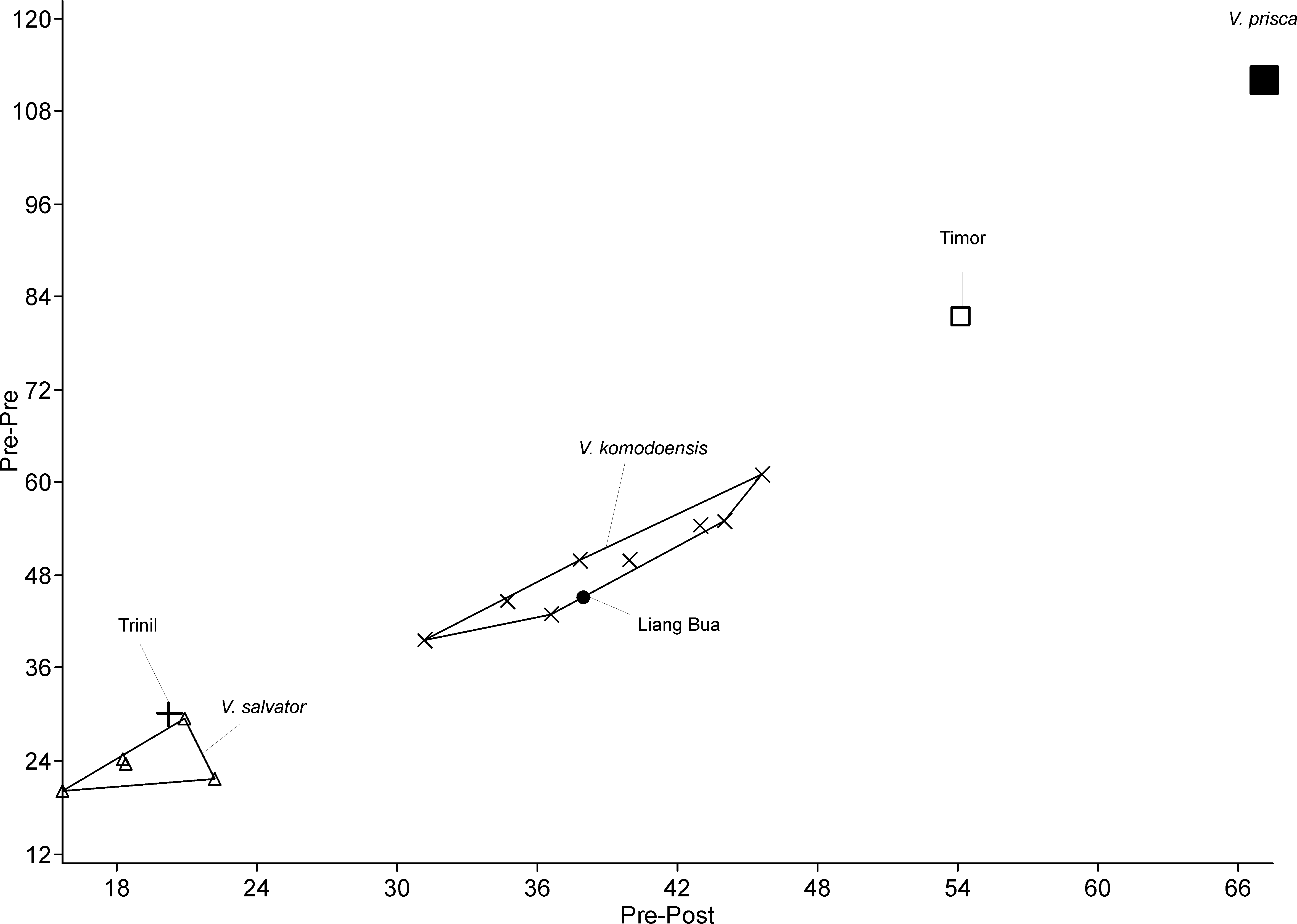


B.


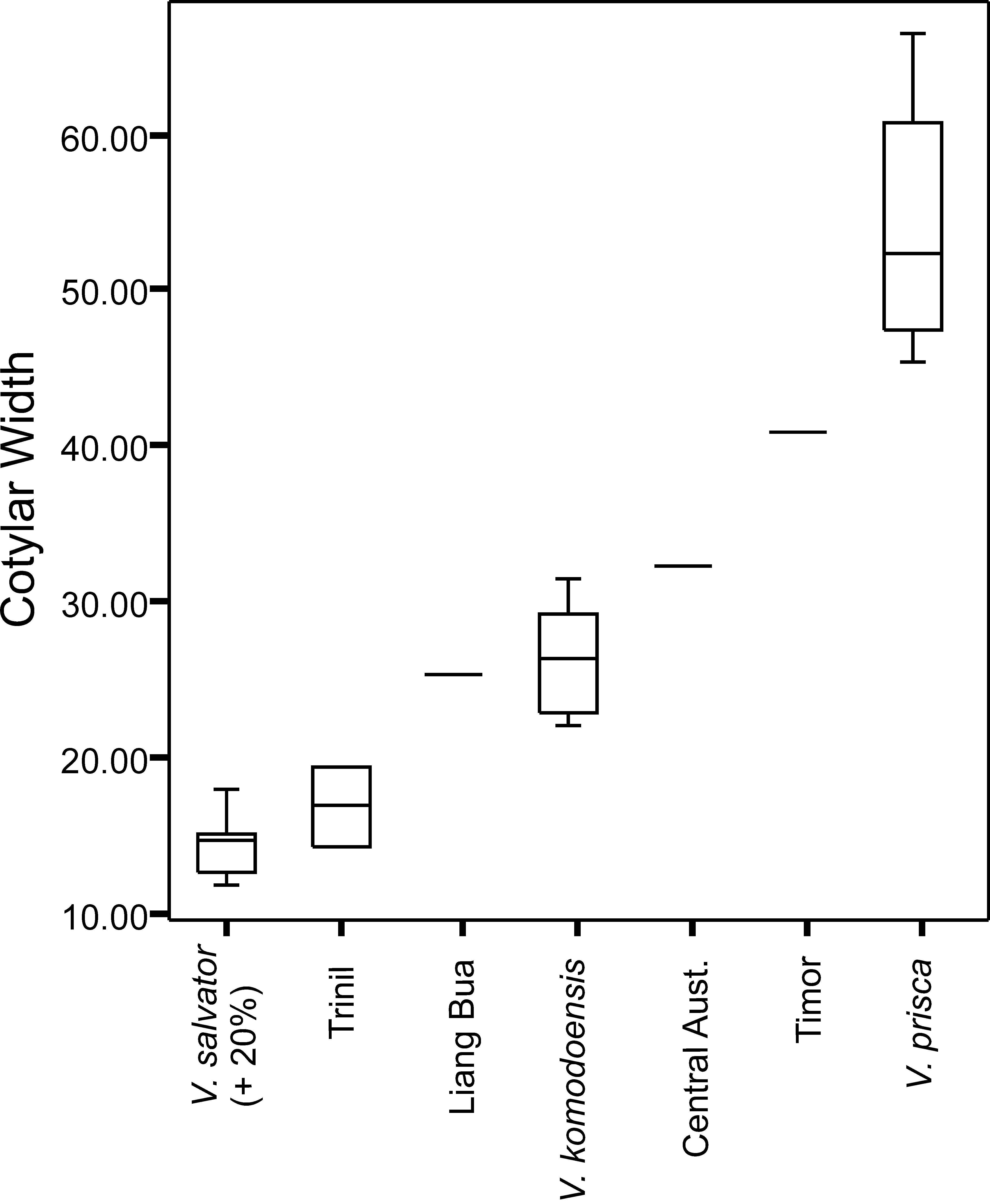


Figure S8. Measurements of varanid sacral vertebrae. A. Bivariate plot of pre-pre length vs pre-post length. Convex hulls applied to show limits of sample variation. B. Box-plot of sacral vertebrae cotylar width measurements. *Varanus salvator* (n = 10), Trinil (n = 2), *Varanus komodoensis* (n = 9), *V. prisca* (n = 4). Measurements in mm.
