# Supplementary material for: Dragon's Paradise Lost: Palaeobiogeography, Evolution and Extinction of the Largest-Ever Terrestrial Lizards (Varanidae)
Source: PLoS One. 2009 Sep 30;4(9):e7241. doi: 10.1371/journal.pone.0007241 (PMC2748693; doi:10.1371/journal.pone.0007241)
Supplement: Figure S9 — Box-plot of caudal vertebrae prezygapophysis-postzygapophysis length measurements. Varanus salvator (n = 9), Varanus komodoensis (n = 24), Liang Bua (n = 4), V. prisca (n = 8). Measurements in mm. (0.06 MB DOC) [file pone.0007241.s009.doc]

Figure S9.


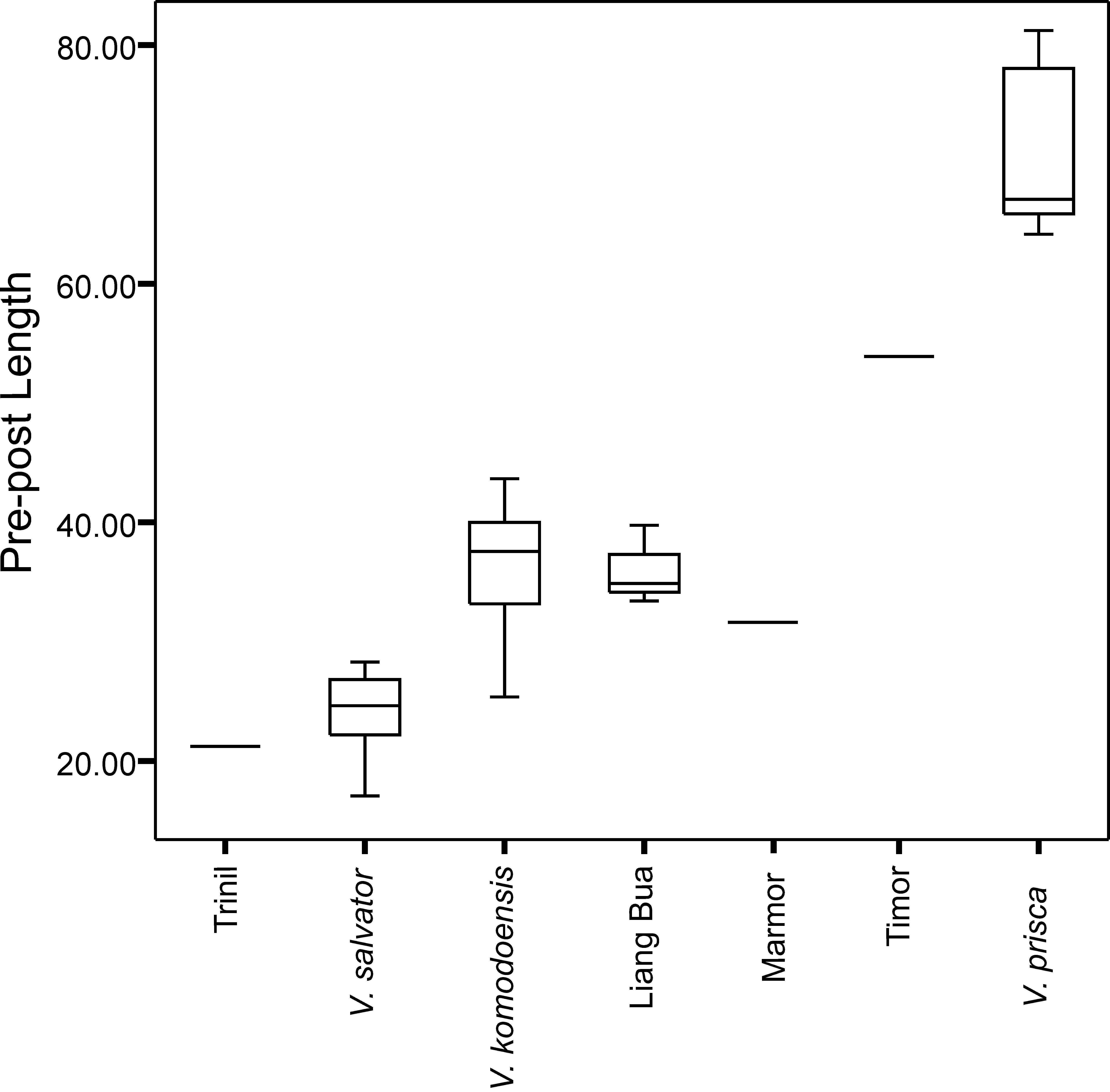


Figure S9. Box-plot of caudal vertebrae prezygapophysis-postzygapophysis length measurements. *Varanus salvator* (n = 9), *Varanus komodoensis* (n = 24), Liang Bua (n = 4), *V. prisca* (n = 8). Measurements in mm.
