# Supplementary material for: Dragon's Paradise Lost: Palaeobiogeography, Evolution and Extinction of the Largest-Ever Terrestrial Lizards (Varanidae)
Source: PLoS One. 2009 Sep 30;4(9):e7241. doi: 10.1371/journal.pone.0007241 (PMC2748693; doi:10.1371/journal.pone.0007241)
Supplement: Figure S10 — Box plot of dorsal vertebra pre-postzygapophysis length for V. prisca (n = 53), Varanus sp. nov. (n = 11) and modern V. komodoensis (n = 32). Measurements in mm. (0.04 MB DOC) [file pone.0007241.s010.doc]

Figure S10.


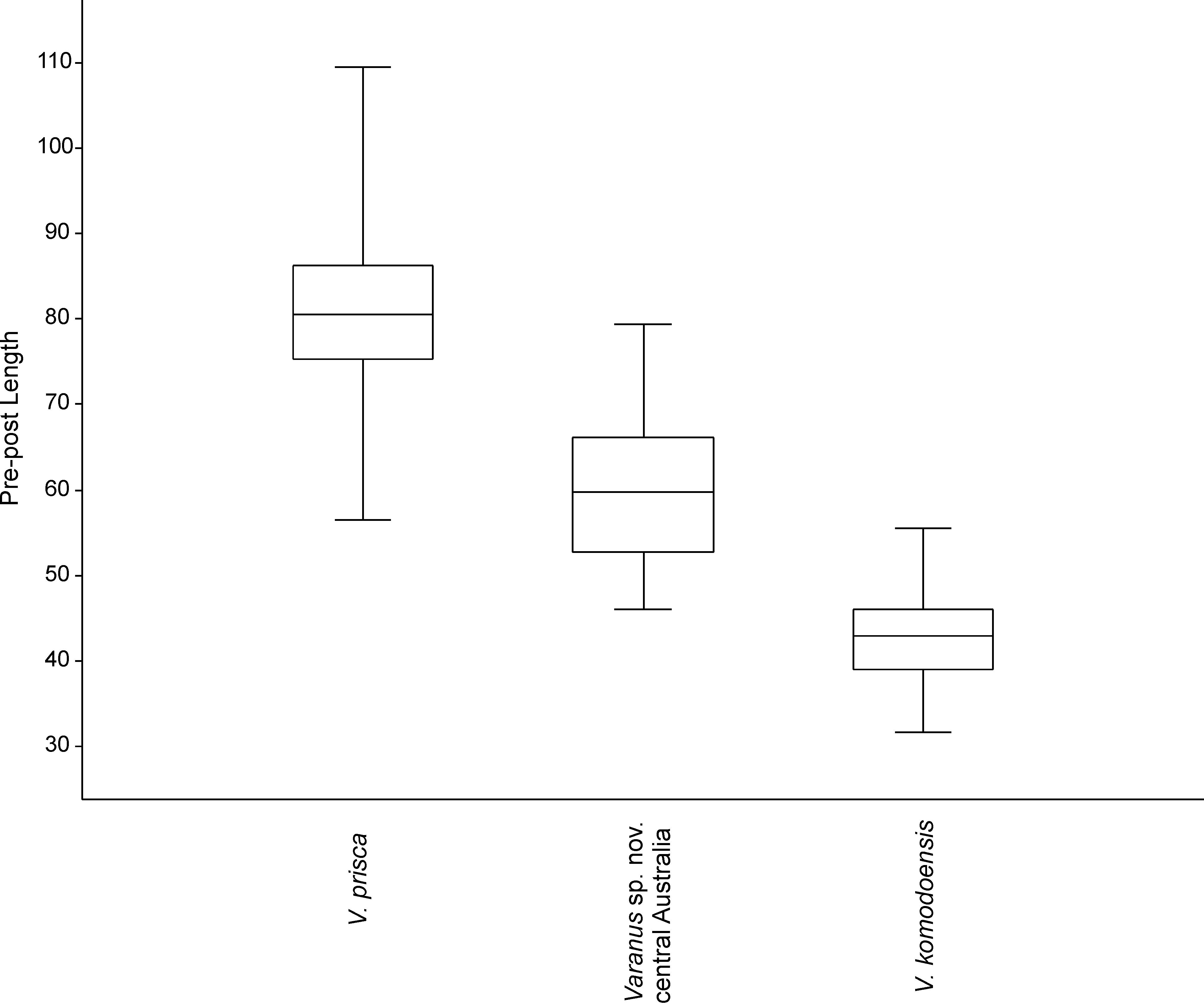


Figure S10. Box plot of dorsal vertebra pre-postzygapophysis length for *V. prisca* (n= 53), *Varanus* sp. nov. (n = 11) and modern *V. komodoensis* (n= 32). Measurements in mm.
