# Supplementary material for: Dragon's Paradise Lost: Palaeobiogeography, Evolution and Extinction of the Largest-Ever Terrestrial Lizards (Varanidae)
Source: PLoS One. 2009 Sep 30;4(9):e7241. doi: 10.1371/journal.pone.0007241 (PMC2748693; doi:10.1371/journal.pone.0007241)
Supplement: Table S1 — Specimens used in this study. (0.28 MB DOC) [file pone.0007241.s012.doc]

**Table S1. Specimens used in this study.**

| **Taxon** | **Specimen No.** | **Specimen Description** | **Age** | **Locality / Formation** | **Province / Country** |
| --- | --- | --- | --- | --- | --- |
| *V. komodoensis* | LB 1-9-03 | Dorsal vertebra | Late Pleistocene | Liang Bua | Flores |
| *V. komodoensis* | LB 485 | Dorsal vertebra | Late Pleistocene | Liang Bua | Flores |
| *V. komodoensis* | LB19/20-8-04 | Dorsal vertebra | Late Pleistocene | Liang Bua | Flores |
| *V. komodoensis* | LB244 | Dorsal vertebra | Late Pleistocene | Liang Bua | Flores |
| *V. komodoensis* | LB298 | Dorsal vertebra | Late Pleistocene | Liang Bua | Flores |
| *V. komodoensis* | LB307 | Dorsal vertebra | Late Pleistocene | Liang Bua | Flores |
| *V. komodoensis* | LB31/07/2004 | Dorsal vertebra | Late Pleistocene | Liang Bua | Flores |
| *V. komodoensis* | LB413 | Dorsal vertebra | Late Pleistocene | Liang Bua | Flores |
| *V. komodoensis* | LB418 | Dorsal vertebra | Late Pleistocene | Liang Bua | Flores |
| *V. komodoensis* | LB420 | Dorsal vertebra | Late Pleistocene | Liang Bua | Flores |
| *V. komodoensis* | LB447 | Dorsal vertebra | Late Pleistocene | Liang Bua | Flores |
| *V. komodoensis* | LB468 | Dorsal vertebra | Late Pleistocene | Liang Bua | Flores |
| *V. komodoensis* | LB517 | Dorsal vertebra | Late Pleistocene | Liang Bua | Flores |
| *V. komodoensis* | LB623 | Dorsal vertebra | Late Pleistocene | Liang Bua | Flores |
| *V. komodoensis* | LB751 | Dorsal vertebra | Late Pleistocene | Liang Bua | Flores |
| *V. komodoensis* | LB398 | Caudal vertebra | Late Pleistocene | Liang Bua | Flores |
| *V. komodoensis* | LB503c, 18-8-04 | Caudal vertebra | Late Pleistocene | Liang Bua | Flores |
| *V. komodoensis* | LB18/08/2004 | Caudal vertebra | Late Pleistocene | Liang Bua | Flores |
| *V. komodoensis* | LB117d | Caudal vertebra | Late Pleistocene | Liang Bua | Flores |
| *V. komodoensis* | LB21.8.03 | Caudal vertebra | Late Pleistocene | Liang Bua | Flores |
| *V. komodoensis* | LB558b | Caudal vertebra | Late Pleistocene | Liang Bua | Flores |
| *V. komodoensis* | LB503d | Cervical vertebra | Late Pleistocene | Liang Bua | Flores |
| *V. komodoensis* | LB517b | Cervical vertebra | Late Pleistocene | Liang Bua | Flores |
| *V. komodoensis* | LB503b | Cervical vertebra | Late Pleistocene | Liang Bua | Flores |
| *V. komodoensis* | TT4062 | Cervical vertebra | Early Pleistocene | Tangi Talo | Flores |
| *V. komodoensis* | LB558a | Sacral vertebra | Late Pleistocene | Liang Bua | Flores |
| *V. komodoensis* | no No. | Tooth | Late Pleistocene | Liang Bua | Flores |
| *V. komodoensis* | no No. | Tooth | Late Pleistocene | Liang Bua | Flores |
| *V. komodoensis* | no No. | 4 x teeth | Late Pleistocene | Liang Bua | Flores |
| *V. komodoensis* | TT-3854A | Tooth | Early Pleistocene | Tangi Talo | Flores |
| *V. komodoensis* | TT-3854B | Tooth | Early Pleistocene | Tangi Talo | Flores |
| *V. komodoensis* | TT-3854C | Tooth | Early Pleistocene | Tangi Talo | Flores |
| *V. komodoensis* | TT-3854D | Tooth | Early Pleistocene | Tangi Talo | Flores |
| *V. komodoensis* | TT-3854E | Tooth | Early Pleistocene | Tangi Talo | Flores |
| *V. komodoensis* | TT-3854F | Tooth | Early Pleistocene | Tangi Talo | Flores |
| *V. komodoensis* | TT-4097 | right humerus diaphysis | Early Pleistocene | Tangi Talo | Flores |
| *V. komodoensis* | LB-28.7.03 | radius diaphysis | Late Pleistocene | Liang Bua | Flores |
| *V. komodoensis* | LB-289a/10.5.01 | 2 ilium fragments of 1 individual | Late Pleistocene | Liang Bua | Flores |
| *V. komodoensis* | LB-578 | 2 metapodials and 1 phalanx | Late Pleistocene | Liang Bua | Flores |
| *V. komodoensis* | LB-19.7.04 | right mandible | Late Pleistocene | Liang Bua | Flores |
| *V. komodoensis* | LB-447a/16.8.04 | left ulna diaphysis | Late Pleistocene | Liang Bua | Flores |
| *V. salvator* | CD1908.3 | Dorsal vertebra | Early Pleistocene | Trinil | Java |
| *V. salvator* | CD207 | Dorsal vertebra | Early Pleistocene | Trinil | Java |
| *V. salvator* | CD3 | Dorsal vertebra | Early Pleistocene | Trinil | Java |
| *V. salvator* | CD5676 | Dorsal vertebra | Early Pleistocene | Trinil | Java |
| *V. salvator* | CD5731 | Dorsal vertebra | Early Pleistocene | Trinil | Java |
| *V. salvator* | CD5782 | Dorsal vertebra | Early Pleistocene | Trinil | Java |
| *V. salvator* | CD5793 | Dorsal vertebra | Early Pleistocene | Trinil | Java |
| *V. salvator* | CD5835 | Dorsal vertebra | Early Pleistocene | Trinil | Java |
| *V. salvator* | CD5917 | Dorsal vertebra | Early Pleistocene | Trinil | Java |
| *V. salvator* | CD6384 | Dorsal vertebra | Early Pleistocene | Trinil | Java |
| *V. salvator* | CD6877 | Dorsal vertebra | Early Pleistocene | Trinil | Java |
| *V. salvator* | CD6902 | Dorsal vertebra | Early Pleistocene | Trinil | Java |
| *V. salvator* | CD6904 | Dorsal vertebra | Early Pleistocene | Trinil | Java |
| *V. salvator* | CD8873 | Dorsal vertebra | Early Pleistocene | Trinil | Java |
| *V. salvator* | CD11194 | Dorsal vertebra | Early Pleistocene | Trinil | Java |
| *V. salvator* | CD6189 | Caudal vertebra | Early Pleistocene | Trinil | Java |
| *V. salvator* | CD8432 | Sacral vertebra | Early Pleistocene | Trinil | Flores |
| *V. salvator* | CD5742 | Sacral vertebra | Early Pleistocene | Trinil | Flores |
| *V. sivalensis (=V. salvator)* | NHMR740 | Dorsal vertebra | Pliocene / Early Pleistocene | Siwalik Hills | India |
| *V. sivalensis (=V. salvator)* | NHMR739 | Cervical vertebra (= Dorsal vertebra) | Pliocene / Early Pleistocene | Siwalik Hills | India |
| *V. sivalensis* | NHMR40819 | Distal Humerus | Pliocene / Early Pleistocene | Siwalik Hills | India |
| *Varanus prisa* | QM & AM Collections | Cranial & post cranial specimens | Late Pleistocene | Eastern Darling Downs | Australia |
| *Varanus prisca* | QM & MOV Collections | Cranial & post cranial specimens | Middle-Late Pleistocene | Wyandotte Fmn | Australia |
| *Varanus prisca* | AM Collections | Postcranial specimens | Late Pleistocene | Cuddie Springs | Australia |
| *Varanus* sp. nov*.* | SAM & UCMP Collection | Postcranial specimens | Middle-Late Pleistocene | Katapiri / Kutjitara Fmn | Australia |
| *Varanus* sp. nov*.* | CV Collection, NNM | Dorsal vertebra | >Middle Pleistocene | Raebia | Timor |
| *Varanus* sp. nov*.* | CV Collection, NNM | Sacral vertebra | >Middle Pleistocene | Raebia | Timor |
| *Varanus* sp. nov*.* | CV Collection, NNM | Caudal vertebra | >Middle Pleistocene | Raebia | Timor |
| *V. komodoensis* | QM Collection | Numerous vertebrae | late Early Pliocene | Chinchilla | Australia |
| *V. komodoensis* | QMF874 | right maxilla | late Early Pliocene | Chinchilla | Australia |
| *V. komodoensis* | QMF870+871 | left mandible | late Early Pliocene | Chinchilla | Australia |
| *V. komodoensis* | QMF866 | right scapulocoracoid | late Early Pliocene | Chinchilla | Australia |
| *V. komodoensis* | QMF42156 | left quadrate | late Early Pliocene | Chinchilla | Australia |
| *V. komodoensis* | QMF53956 | parietal | late Early Pliocene | Chinchilla | Australia |
| *V. komodoensis* | QMF53955 | right humerus | late Early Pliocene | Chinchilla | Australia |
| *V. komodoensis* | QMF53954 | left humerus | late Early Pliocene | Chinchilla | Australia |
| *V. komodoensis* | QMF42105 | right maxilla | late Early Pliocene | Chinchilla | Australia |
| *V. komodoensis* | QMF 25392 | supraorbital | Pliocene | Chinchilla | Australia |
| *V. komodoensis* | QM23686 | Dorsal vertebra | Early Pliocene | Bluff Downs | Australia |
| *V. komodoensis* | QMF23684 | Cervical vertebra | Early Pliocene | Bluff Downs | Australia |
| *V. komodoensis* | QMF 54605 | Maxilla | Middle Pleistocene | Mt. Etna | Australia |
| *V. komodoensis* | QMF 54607 | Supraocciptial | Middle Pleistocene | Mt. Etna | Australia |
| *V. komodoensis* | QMF 54606 | Quadrate | Middle Pleistocene | Mt. Etna | Australia |
| *V. komodoensis* | QMF 54608 | Tibia | Middle Pleistocene | Mt. Etna | Australia |
| *V. komodoensis* | QMF 54604 | Ulna | Middle Pleistocene | Mt. Etna | Australia |
| *V. komodoensis* | QMF54120 | Dorsal vertebra | Middle Pleistocene | Mt. Etna | Australia |
| *V. komodoensis* | QMF 1418 | Caudal vertebra | Middle Pleistocene | Marmor Quarry | Australia |
| *Varanus.* cf. *V. komodoensis* | CD6392 | Dorsal vertebra | Middle Pleistocene | Kedung Brubus | Java |
|  |  |  |  |  |  |
| **Comparative Specimens** |  |  |  |  |  |
| *Varanus komodoensis* | NNM17504 | Skeleton | Modern | Flores | Indonesia |
| *Varanus komodoensis* | NNM17494 | Skeleton | Modern | Rinca | Indonesia |
| *Varanus komodoensis* | NNM6994 | Skeleton | Modern | West Flores | Indonesia |
| *Varanus komodoensis* | NNM21-11-38 | Skeleton | Modern | ? | Indonesia |
| *Varanus komodoensis* | NNM35517 | Skeleton | Modern | ? | Indonesia |
| *Varanus komodoensis* | NNM35518 | Skeleton | Modern | ? | Indonesia |
| *Varanus komodoensis* | NNM35515 | Skeleton | Modern | ? | Indonesia |
| *Varanus komodoensis* | NNM35510 | Skeleton | Modern | Komodo | Indonesia |
| *Varanus komodoensis* | USNM228163 | Skeleton | Modern | ? | Indonesia |
| *Varanus komodoensis* | USNM101444 | Skeleton | Modern | ? | Indonesia |
| *Varanus komodoensis* | NHM934.9.2 | Skeleton | Modern | Komodo | Indonesia |
| *Varanus komodoensis* | USNM220286 | Skeleton | Modern | ? | Indonesia |
| *Varanus komodoensis* | USNM220287 | Skeleton | Modern | ? | Indonesia |
| *Varanus komodoensis* | USNM221892 | Skeleton | Modern | ? | Indonesia |
| *Varanus komodoensis* | LACM | Skeleton | Modern | ? | Indonesia |
| *Varanus salvator* | USNM220287 | Skeleton | Modern | Borneo | Indonesia |
| *Varanus salvator* | NAU QSP | Skeleton | Modern | ? | Indonesia |
| *Varanus salvator* | NAU QSP | Skeleton | Modern | ? | Indonesia |
| *Varanus salvator* | NNM9505906 | Skeleton | Modern | West Java | Indonesia |
| *Varanus salvator* | NHM64.92.77 | Skeleton | Modern | Borneo | Indonesia |
| *Varanus salvator* | NHMB1972 | Skeleton | Modern | Ceylon | Indonesia |
| *Varanus salvator* | NHM2160 | Skeleton | Modern | Ceylon | Indonesia |
| *Varanus salvator* | NHM?314 | Skeleton | Modern | ? | ? |
| *Varanus salvator* | NHM17.4.42 | Skeleton | Modern | Ceylon | Indonesia |
| *Varanus salvadorii* | QMJ14498 | Skeleton | Modern | PNG | PNG |
| *Varanus* spp. | QM Collection | Skeletons | Modern | Various | Australia |
|  |  |  |  |  |  |
| **Collection Abbreviations** |  |  |  |  |  |
| **ARKENAS** | National Archaeological Research Centre, Jakarta, Indonesia |  |  |  |  |
| **NHMR** | British Museum of Natural History Fossil |  |  |  |  |
| **NHM** | British Natural History Museum |  |  |  |  |
|  | Extant |  |  |  |  |
| **CD** | Dubois Collection, NNM |  |  |  |  |
| **CV** | Verhoeven Collection, NNM |  |  |  |  |
| **NNM** | Naturalis |  |  |  |  |
|  | National Museum of Natural History |  |  |  |  |
|  | The Netherlands |  |  |  |  |
| **LB** | Liang Bua Collection, ARKENAS |  |  |  |  |
| **TT** | Tangi Talo Collection, GSI |  |  |  |  |
| **GSI** | Geological Survey Institute, Bandung Indonesia (formerly GRDC) |  |  |  |  |
|  |  |  |  |  |  |
|  |  |  |  |  |  |
| **USNM** | United States National Museum |  |  |  |  |
|  | Smithsonian Institute |  |  |  |  |
|  | Washington, United States of America |  |  |  |  |
| **NAU** | Northern Arizona University |  |  |  |  |
|  | Quaternary Sciences Program Collection |  |  |  |  |
|  | Flagstaff, United States of America |  |  |  |  |
| **UCMP** | University of California Museum of Palaeontology Collection, Berkeley |  |  |  |  |
|  | California, United States of America |  |  |  |  |
| **QMF** | Queensland Museum Fossil |  |  |  |  |
|  |  |  |  |  |  |
| **SAM** | South Australian Museum |  |  |  |  |
|  | Adelaide |  |  |  |  |
| **AM** | Australian Museum |  |  |  |  |
|  | Sydney |  |  |  |  |
| **MOV** | Museum of Victoria |  |  |  |  |
|  | Melbourne |  |  |  |  |
|  | Australia |  |  |  |  |
